# Replication Stress Defines Distinct Molecular Subtypes Across Cancers

**DOI:** 10.1158/2767-9764.CRC-22-0168

**Published:** 2022-06-24

**Authors:** Nobuyuki Takahashi, Sehyun Kim, Christopher W. Schultz, Vinodh N. Rajapakse, Yang Zhang, Christophe E. Redon, Haiqing Fu, Lorinc Pongor, Suresh Kumar, Yves Pommier, Mirit I. Aladjem, Anish Thomas

**Affiliations:** 1 Developmental Therapeutics Branch, Center for Cancer Research, NCI, Bethesda, Maryland.; 2 Medical Oncology Branch, Center Hospital, National Center for Global Health and Medicine, Tokyo, Japan.; 3 Department of Medical Oncology, National Cancer Center East Hospital, Chiba, Japan.; 4 Department of Internal Medicine, Seoul National University Bundang Hospital, Seoul National University College of Medicine, Seongnam, Korea.

## Abstract

Endogenous replication stress is a major driver of genomic instability. Current assessments of replication stress are low throughput precluding its comprehensive assessment across tumors. Here we develop and validate a transcriptional profile of replication stress by leveraging established cellular characteristics that portend replication stress. The repstress gene signature defines a subset of tumors across lineages characterized by activated oncogenes, aneuploidy, extrachromosomal DNA amplification, immune evasion, high genomic instability, and poor survival, and importantly predicts response to agents targeting replication stress more robustly than previously reported transcriptomic measures of replication stress. Repstress score profiles the dual roles of replication stress during tumorigenesis and in established cancers and defines distinct molecular subtypes within cancers that may be more vulnerable to drugs targeting this dependency. Altogether, our study provides a molecular profile of replication stress, providing novel biological insights of the replication stress phenotype, with clinical implications.

**Significance:**

We develop a transcriptional profile of replication stress which characterizes replication stress and its cellular response, revealing phenotypes of replication stress across cancer types. We envision the repstress score to serve as an effective discovery platform to predict efficacy of agents targeting replication stress and clinical outcomes.

## Introduction

Genomic instability is an enabling characteristic of cancer, which by generating genetic diversity expedites the acquisition of multiple hallmark capabilities (1). DNA damage resulting from unabated replication—referred to as replication stress—is a major driver of genomic instability (2). Cells have evolved multiple mechanisms to sense and respond to replication stress, together referred to as the replication stress response (3). When replication fork stalls, the exposed single-stranded DNA (ssDNA) is rapidly coated by ssDNA-binding proteins such as replication protein-A (RPA), leading to activation of ataxia telangiectasia and Rad3-related kinase (ATR), which subsequently phosphorylates downstream kinases including CHK1 (4). ATR and CHK1 negatively regulate cyclin-dependent kinase (CDK) activity through phosphorylation of WEE1 and other substrates. ATR also delays exhaustion of RPA and global breakage of active forks by limiting origin firing (5). Together, the replication stress response cascade prevents stalling of replication forks, controls the initiation of DNA replication, ensures sufficient supply of nucleotides, and limits mitotic entry of cells that have not yet completed DNA replication. Failure to resolve replication stress can lead to collapse of replication forks, DNA double-strand breaks, and acquisition of mutations that are deleterious to genome integrity (2).

Replication stress is a feature of precancerous (6) and cancerous cells (7). Cancer cells exhibit heightened replication stress response, for example through *CHEK1* amplification, to support rapid proliferation and tolerate the higher levels of replication stress (8). Replication stress itself and the mechanisms that mitigate replication stress are increasingly recognized as cancer cell–specific vulnerabilities that could be exploited therapeutically (9–12). However, rational targeting of these dependencies requires reliable approaches to assess replication stress and its cellular response in patient tumors. Measures of replication stress—including ssDNA or ssDNA-bound RPA levels, phosphorylated form of histone H2AX (γH2AX)—are widely used in experimental settings (13, 14), but are not optimized for use in large cohorts of clinical tumor samples. Here we develop and validate a transcriptional profiling–based approach—the repstress gene signature—that characterizes the cellular response to replication stress at a functional network level (Supplementary Fig. S1).

## Materials and Methods

### Data Acquisition

RNA sequencing (RNA-seq), mutations, copy-number states, drug activity, and doubling time in NCI Development Therapeutics Program small cell lung cancer (NCI-DTP SCLC), Cancer Cell Line Encyclopedia (CCLE), Genomics of Drug Sensitivity in Cancer, Cancer Therapeutics Response Portal (CTRP), and NCI60 were downloaded from CellMiner CDB (15, 16). Clinical, pathologic, and molecular characteristics, survival, RNA-seq, expression of reverse phase protein array (RPPA), genomic alteration, and copy-number alteration for The Cancer Cell Genome Atlas (TCGA) samples were retrieved from data hub of Pan-Cancer TCGA dataset in University of California Santa Cruz Xena platform (17). For other dataset used in this study, please refer Supplementary Text in Supplementary Materials and Methods.

### Development of Repstress Gene Signature

To develop repstress gene signature, we focused on four biological characteristics associating with replication stress in SCLC cell lines: *MYC*-paralog genes amplification, sensitivity to cell-cycle checkpoint inhibitors, high expression of phosphorylated Chk1 (p-Chk1), and neuroendocrine (NE) differentiation. We defined *MYC*-amplified SCLC cell lines using the cutoff of 0.7 or more of copy-number score (the average log_2_-transformed probe intensity ratio of gene specific chromosomal segment DNA relative to normal DNA) in either of *MYC* family genes (*MYC*, *MYCL*, *MYCN*). Cell-cycle checkpoint inhibitor–sensitive SCLC cell lines were defined as those with drug activity score [standardized, z-score normalized measurements provided from the mean and SD of −log_10_ (molar concentration causing 50% cell growth inhibition, GI_50_) values over NCI-DTP SCLC cell lines] of more than 6 with CHK1 inhibitor AZD-7762 (drug ID: 754352) or WEE1 inhibitor MK-1775 (drug ID: 757148). For details of these scores, please refer a previous report describing methods used in CellMiner CDB (16). High expression of p-Chk1 was defined as Chk1_pS345 RPPA expression of more than 0.15. We subsequently applied gene set enrichment analysis (GSEA) using Hallmark gene sets (18) comparing differentially regulated pathways between SCLC cell lines with one of these characteristics and those without. By using adjusted *P* value of <0.05, we identified two shared hallmark gene sets (HALLMARK_E2F_TARGET and HALLMARK_G2M_CHECKPOINT) as commonly upregulated pathways in SCLC cell lines with one of the repstress characteristics across all of the hallmark genesets. During the GSEA, 11 genes (*AURKB*, *CCNA2*, *GINS1*, *KPNA2*, *LIG3*, *MTF2*, *ORC6*, *PRPS1*, *SRSF1*, *SUV39H1*, *TNPO2*) were found as shared leading-edge genes of the two gene sets. Neuroendocrine status of SCLC cell lines (19) and clinical tumors in an independent cohort (20) were assessed using single-sample GSEA (21) of previously described 50 NE gene set, containing 25 genes associated with high neuroendocrine and 25 genes associated with low NE (22). High-neuroendocrine score and low-neuroendocrine score were calculated by single-sample GSEA separately using each of the 25 high of low NE genes and compared the two scores with define high versus low neuroendocrine differentiated SCLC cell lines (15) and clinical tumors (20). Subsequently, differentially expressing genes were analyzed between high versus low neuroendocrine differentiated SCLC cell lines or tumors in each cohort. Among identified highly expressing genes in neuroendocrine differentiated SCLC, by FDR of <10% by Mann–Whitney *U* test followed by adjusting multiple testing with Benjamini–Hochberg test, those identified in both two cohort and involved in DNA damage repair pathways (23) were defined as additional repstress signature genes (*GADD45G*, *POLA1*, *POLD4*, *POLE4*, *RFC5*, *RMI1*, and *RRM1*). We finally excluded the gene *KPNA2* from the repstress gene signature because it did not frequently express in cell lines other than SCLC (Supplementary Table S1).

Repstress score was calculated by applying principal component analysis–based weighting score. In detail, SCLC cell lines were projected onto principal component analysis plot using the scores for biological characteristics associated with replication stress described above and the 17 repstress gene expression were also projected onto the plot, which achieved variable loadings of first principal component dimension for each gene as gene weight (Supplementary Fig. S2A; Supplementary Table S1). We summed up the measurements of repstress signature gene expressions (Z score–normalized in each cell line across all of sequenced gene expressions) multiplied by each gene weight and defined as repstress score. Repstress scores were Z score–normalized among samples used in each analysis and shown in figures.

### SCLC Cell Lines

Nine SCLC cell lines (NCI-H1048; RRID: CVCL_1453, NCI-H1341; RRID: CVCL_1463, NCI-H841; RRID: CVCL_1595, DMS114; RRID: CVCL_1562, NCI-H211; RRID: CVCL_1529, NCI-H446 RRID: CVCL_1562, NCI-H889: RRID: CVCL_1598, NCI-H146; RRID: CVCL_1473, NCI-H524; RRID: CVCL_1568) were purchased from ATCC and maintained in cell culture. H211, H889, H1048, and H1341 cell lines are female and the rest are male. Cell lines were authenticated using short tandem repeat analysis, and were monthly tested for *Mycoplasma* contamination. Cell media was RPMI1640 supplemented with 10% FBS for all lines to maintain consistency. Cells were grown at 37°C and 5% CO_2_ were used in subsequent experiments.

### Western Blot Analysis

Cells were lysed with RIPA buffer containing protease inhibitor cocktail (Thermo Fisher Scientific) and micrococcal nuclease (Thermo Fisher Scientific). The resulting mixtures were incubated on ice for 30 minutes, then centrifuged 20 minutes to get the supernatants. After adding Tris-Glycine SDS sample buffer including 5% of 2-mercaptoethanol, the lysates were boiled for 10 minutes, analyzed by SDS-PAGE, and immunoblotted with various antibodies as follows: RPA phosphorylation (pS4/8, from Bethyl Laboratories; RRID: AB_2891810); total RPA (from Bethyl Laboratories; RRID: AB_185548); pATR (T1989, from Cell Signaling Technology; RRID:AB_2722679); and pCHK1(S345, from Cell Signaling Technology; RRID:AB_330023). To start Western blot analysis, nitrocellulose membrane was blocked with 5% nonfat milk, then incubated with primary antibodies at 1:1,000 dilution in PBST buffer (PBS containing 0.1% Tween 20) containing 1% nonfat milk, at 4°C overnight. After washing with PBST three times, the membrane was incubated with second antibody at 1:2,000 dilution in PBST buffer containing 1% nonfat milk, at room temperature for 1 hour. The Western blot analysis results were developed by Bio-Rad ChemiDoc MP Imaging System.

### Immunofluorescence Assay

Cells were fixed with 2% paraformaldehyde, followed by the incubation with 70% cold ethanol. After blocking with 5% BSA, primary antibody staining was performed as follows: anti-γH2AX (1:500, Millipore, 05-636), anti-phosphorylated replication protein A (pRPA; 1:500, Bethyl Laboratories, A300-245A; RRID: AB_210547). Secondary antibody staining was performed as follows: Alexa 488–conjugated anti-mouse lgG and Alexa 594–conjugated anti-rabbit lgG (1:500, Cell Signaling Technology, 4408 and 8889). 4′,6-diamidino-2-phenylindole (DAPI) staining was performed with VECTASHIELD mounting medium with DAPI (H-1200, Vector Laboratories). A Zeiss LSM780 confocal microscope was used to capture the fluorescence. The Colocalization Plugin of the FIJI-ImageJ software was used to calculate the fluorescence density.

### 5-ethynyl-2ʹ-deoxyuridine Incorporation and γH2AX Induction Upon Topotecan Treatment

Cell lines were plated at 1 million cells per 10-cm plate. After 24 hours, cells were treated for 2 hours with either DMSO control or 10 μmol/L topotecan, and for 1 hour (the second hour of topotecan treatment) with 1 μmol/L 5-ethynyl-2′-deoxyuridine (EdU). Cells were fixed in and stained for γH2AX as described previously (24), followed by Click-iT Chemistry as per manufacturer's instructions utilizing the Click-iT Plus EdU Alexa Fluor 647 Flow Cytometry Assay Kit C10634 (Thermo Fisher Scientific). Flow cytometry data were collected using a BD LSRFortesa and analyzed utilizing FlowJo V10.7.1.

### DNA Combing Analysis

As described previously (25), asynchronous DMS114 and H524 cells were sequentially labeled with 20 μmol/L IdU for 20 minutes and 50 μmol/L CldU for 20 minutes. To preserve long genomic DNA fibers, cells were embedded in low melting point agarose plugs and incubated in cell lysis buffer with proteinase K at 50°C overnight. Washed plugs with TE buffer, and then melted plugs in 0.1 mol/L MES (pH 6.5) at 70°C for 20 minutes. Agarose was subsequently degraded by adding 2 μL of β-agarase (New England Biolabs). DNA fibers were then stretched onto salinized coverslips (Genomic Vision, cov-002-RUO) using an in-house combing machine. Combed DNA on coverslips was then baked at 60°C for 2 hours and denatured in 0.5 N NaOH for 20 minutes. IdU, CldU, and ssDNA were detected using a mouse antibody directed against BrdU (IgG1, Becton Dickinson, 347580, 1:25 dilution), a rat antibody directed against BrdU (Accurate Chemical, OBT0030, 1:200 dilution), and a mouse antibody directed against ssDNA (IgG 2a, Millipore, MAB3034, 1:100), respectively. The secondary antibodies used were goat anti-mouse Cy3 (Abcam ab6946), goat anti-rat Cy5 (Abcam, ab6565), and goat anti-mouse BV480 (Jackson ImmunoResearch, 115-685-166) for ssDNA. Slides were scanned with a Fiber-Vision Automated Scanner (Genomic Vision). Replication signals on single DNA fibers were analyzed using FiberStudio (Genomic Vision).

### Graph Generation and Statistical Analysis

All figures were generated using CellMiner CDB (16), GraphPad PRISM software version 8.1.2 (GraphPad Software), R version 1.2.135 (R Foundation for Statistical Computing), and STATA software version 16.0 (StataCorp). Box plots in this article were shown by Tukey box and whisker plots, unless specifically indicated in figure legends. Methods for statistical analyses were indicated in the article and figure legends and were performed using softwares described above. Overall survival (OS) curves were created by the Kaplan–Meier method and compared by log-rank test. All statistical tests were two sided.

### Data Availability

The data analyzed in this study were obtained from public database. The experimental data generated in this study are available upon request from the corresponding author.

## Results

### Development and Validation of a Replication Stress Response Signature

While replication stress is widely prevalent across cancers, it is more central to the tumorigenesis of some cancers than others (7). We chose to develop a replication stress response signature in SCLC, a fast-growing and deadly cancer with molecular and clinical features distinct from other lung cancers. We reasoned that signatures that report replication stress response in SCLC could then be extended to other tumors that also exhibit this phenotype.

SCLCs are characterized by high degree of genomic instability, an important consequence of replication stress (26). Nearly all SCLCs have loss-of-function alterations in tumor suppressors *RB1* and *TP53*, and frequently exhibit amplification and overexpression of oncogenes such as *MYC* (20). SCLCs also exhibit sustained high expression of lineage transcription factors, which contribute to replication stress (27), and are highly vulnerable to perturbation of the transcriptional state (28, 29). Not surprisingly, the standard treatment of SCLC consists mostly of DNA-damaging agents such as platinum compounds, topoisomerase I and II inhibitors, and an alkylating agent temozolomide.

**FIGURE 1:**
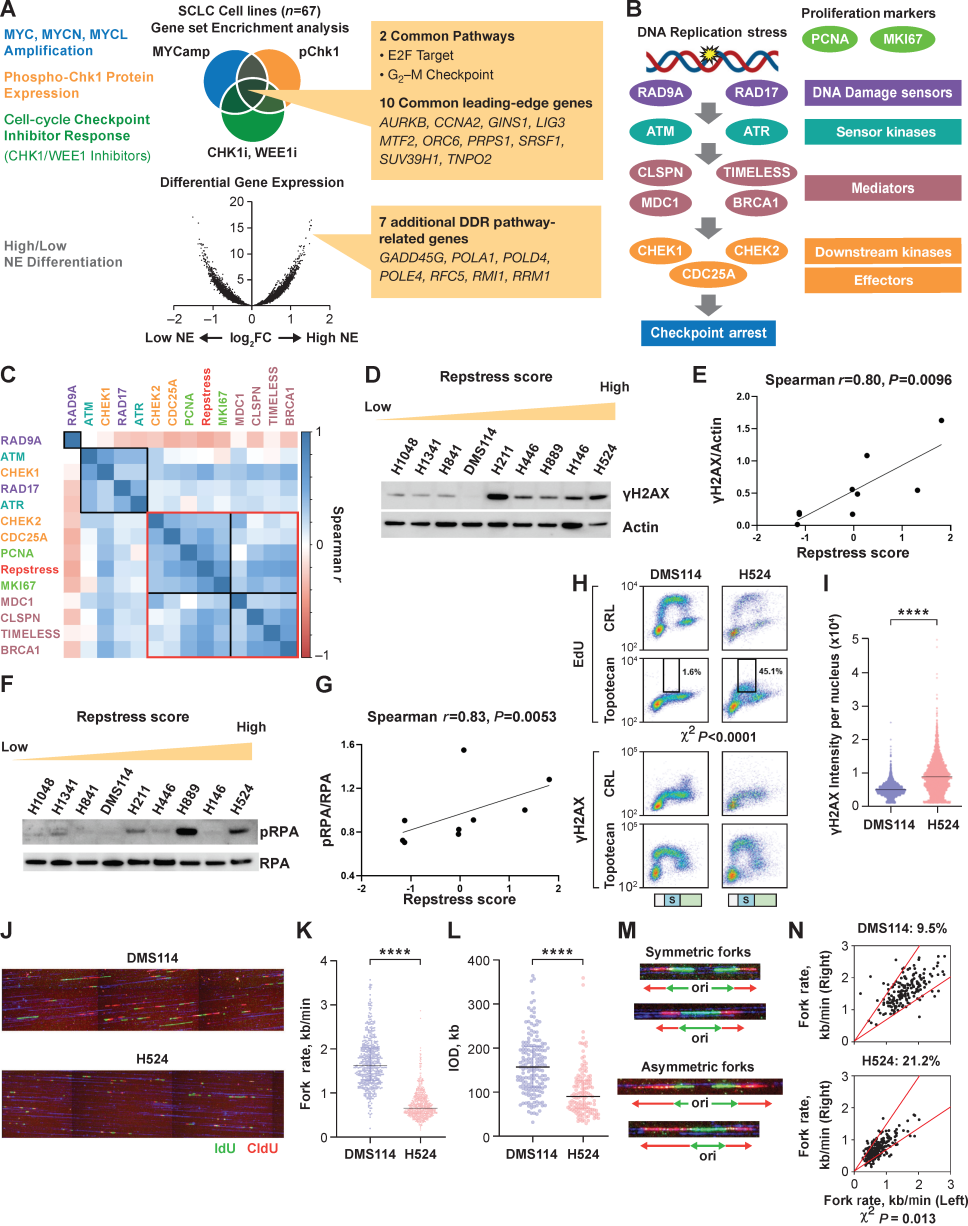
Generation and *in vitro* functional validation of a repstress gene signature in SCLC cell lines. **A,** Schematic representation of the repstress gene signature derivation, which is based on four key characteristics associated with replication stress: (i) amplification of MYC paralogs; (ii) expression of p-Chk1; (iii) sensitivity with CHK1 and WEE1 inhibitors; and (iv) NE. **B,** Schematic representation highlighting key components of the replication stress response pathway The DNA damage sensors recruit kinases ATM and ATR that in turn phosphorylate mediators such as MDC1 and BRCA1 which sustain the DDR signaling. DDR signaling then engages downstream kinases CHK1 and CHK2 and eventually activates downstream effectors such as CDC25A phosphatases triggering transient cell-cycle arrest. **C,** Pairwise correlations between expression of DDR genes, proliferation markers *PCNA* and *MKI67*, and repstress score in 67 SCLC cell lines. Colors of gene name labels denote replication stress response functions indicated in **B**. Genes are clustered by Euclidean distance, using the complete-linkage clustering method, indicated with squares with black and red lines. Western blot analysis (**D**) and correlations (**E**) of γH2AX signal with repstress score in SCLC cell lines. SCLC cell lines are ordered from low to high repstress score (range: −1.2 to 1.8) from left to right in **D**. Western blot analysis (**F**) and correlations (**G**) of pRPA signal with repstress score in SCLC cell lines. SCLC cell lines are ordered from low to high repstress score (range: −1.2 to 1.8) from left to right in **F**. **H** and **I,** S-phase arrest and induction of γH2AX by exogenous replication stress by topotecan treatment in S-phase SCLC cell lines. EdU incorporation (top) and γH2AX induction (bottom) in SCLC cell lines with low (DMS114) and high repstress score (H524) are shown in **H**. Cell-cycle effects are defined by propidium iodide staining (Supplementary Fig. S5) and G_1_, S, G_2_–M phases are indicated on the bottom of the panels with light green, light blue with the letter of S, and light orange bars, respectively. Black squares indicate proportion of EdU incorporating S-phase cells, gated by cutoff of EdU signal intensity >1.0 × 10^3^. A comparison of quantified γH2AX signal intensity per nucleus with topotecan treatment in S-phase cells is shown in **I**. ****, *P* < 0.0001 by unpaired Student *t* test. **J–L,** DNA combing analysis of SCLC cell lines with low (DMS114) and high (H524) repstress scores. Representative images (**J**) and quantifications of replication fork speed (**K**) and interorigin distance (**L**) are shown. Green and red lines in **J** indicate IdU and CIdU, respectively. ****, *P* < 0.0001 by Mann–Whitney *U* test. Representative images (**M**) and quantification (**N**) of fork asymmetry in DNA combing analysis of SCLC cell lines with low (DMS114) and high (H524) repstress score Fork asymmetry was defined by >30% difference of fork speed between one direction with the other as described previously (25), indicating with a redline in **N**. The proportions of DNAs with fork asymmetry in each cell line were indicated on top of **N**. MYCamp, MYC amplification; WEEi1, WEE1 inhibitor; CHK1i, CHK1 inhibitor; p-Chk1, phosphorylated Chk1; Cont, control; PI, propidium iodide; CIdU, chlorodeoxyuridine; kb, kilobase; IOD, interorigin distance; ori, origin.

To obtain a comprehensive molecular understanding of the replication stress response, we examined a panel of 67 SCLC cell lines characterized by microarray-based gene expression, representing the molecular diversity of the disease (15, 19). We reasoned that SCLC cells under high replication stress might be characterized by amplification of *MYC* and its paralogs *MYCN* and *MYCL* (30, 31); expression of p-Chk1 (32); sensitivity to inhibitors of cell-cycle checkpoints CHK1 and WEE1 (33); and NE differentiation (12, 29, 34). GSEA was performed to define differentially regulated biological processes between SCLCs with and without these features, revealing cell cycle–related targets of E2F transcription factors and genes involved in the G_2_–M checkpoint (*AURKB*, *CCNA2*, *GINS1*, *LIG3*, *MTF2*, *ORC6*, *PRPS1*, *SRSF1*, *SUV39H1*, *TNPO2*) and DNA replication and repair genes associated with NE differentiation (*GADD45G*, *POLA1*, *POLD4*, *POLE4*, *RFC5*, *RMI1*, and *RRM1*), together designated as the repstress gene signature (Fig. 1A; Supplementary Table S1). Repstress signature score was calculated using weighted principal component analysis (Supplementary Fig. S2A; Supplementary Table S1), with most genes providing positive signature weightings except *POLD4* and *POLE4*.

Repstress signature included genes involved in mitosis (*AURKB*), cell-cycle progression (*CCNA2*), initiation of replication and replisome progression (*GINS1, ORC6*, *RFC5*), nuclear transport (*TNPO2*), DNA and RNA metabolism (*LIG3*, *PRPS1*, *RMI1*, *RRM1*), transcriptional regulation (*MTF2*, *SUV39H1*), RNA splicing (*SRSF1*), and DNA polymerases (*POLA1*, *POLD4*, *POLE4*). High repstress cells had elevated expression of *MDC1*, *CLSPN*, and *TIMELESS*, genes involved in replication stress tolerance by protecting the replication fork, downstream effectors *CHEK2* and *CDC25A*, and genes associated with proliferation *PCNA* and *MKI67* (ranges of Spearman correlation coefficient and multiple testing *P*_adj_ value: 0.22 to 0.61 and 5.5 × 10^−7^ to 7.7 × 10^−4^, respectively). In contrast, DNA damage sensors *RAD9A* and *RAD17* and sensor kinases *ATM* and *ATR* were less correlated with repstress score (Fig. 1B and C; Supplementary Fig. S2B–D). Repstress score correlated positively with the expression of genes involved in solving topological problems during replication (*TOP2A*), facilitating the repair and restart of stalled replication forks (*FANCD2*), resolving barriers to replication fork progression (*RNASEH2A*), and DNA repair (*POLQ* and *PARP1*; Supplementary Fig. S2E–S2I).

Stalled replication forks require the surrounding chromatin to be compacted for their stabilization (35); the expansion of heterochromatic regions is mediated by histone modifications and attenuates replication stress signaling. We reasoned that if repstress score captures replication stress response at a functional network level, it may be able to predict the heterochromatin response as well. To test this possibility, we examined pairwise correlations between the repstress score and expression of chromatin remodelers and histone modifiers. Repstress score correlated positively with the expressions of heterochromatin proteins HP1α, HP1β, and HP1γ that associate with methylated histone H3 on nucleosomes and mediate heterochromatin formation (ranges of Spearman correlation coefficients and multiple testing *P*_adj_ values: 0.44 to 0.56 and 1.4 × 10^−5^ to 2.8 × 10^−3^, respectively). In contrast, genes involved in INO80 chromatin remodeling complex (*INO80* and *ARP8*) were less correlated with repstress signature and clustered separately (0.11–0.25 and 0.6–1.0, respectively; Supplementary Fig. S2J).

Stressed DNA replication results in DNA double-strand breaks, which induce rapid phosphorylation of H2AX on Ser139, termed as γH2AX. γH2AX is a sensitive albeit indirect indicator of replication stress (36). We detected higher basal endogenous expression of γH2AX by Western blot analysis in SCLC cells with high repstress score compared with cells with low repstress score (Spearman correlation coefficient and *P* value: 0.80 and 0.0096, respectively; Fig. 1D and E). Other replication stress–associated proteins such as phosphorylated RPA, Chk1, and ATR also had positive correlations with repstress score (Fig. 1F and G; Supplementary Fig. S3). Higher basal levels of γH2AX and phosphorylated RPA were also detected by fluorescence microscopy in repstress-high H524 cell line compared with repstress-low DMS114 (Supplementary Fig. S4).

We then assessed whether cells with variable repstress scores responded differentially to exogenous replication stress, using topotecan which produces replication blocks by generating topoisomerase I−DNA cleavage complexes, in two representative cell lines H524 and DMS114 with high and low repstress scores, respectively. At basal levels without drug treatment, H524 cells exhibited lower DNA synthesis and more DNA damage during S-phase, as indicated by the proportion of cells labeled with EdU and γH2AX, respectively, compared with DMS114 cells. Upon treatment with topotecan, DNA synthesis and cell proliferation were inhibited to a much lesser extent in H524 cells compared with DMS114 (Fig. 1H; Supplementary Fig. S5), resulting in higher induction of γH2AX in H524 (Fig. 1H and I). The γH2AX induction by topotecan treatment correlated with the repstress score in a larger panel of SCLC cell lines (Supplementary Fig. S6). To further elucidate the dynamics of DNA replication, we performed DNA combing assay. H524 cells had markedly lower fork velocities and inter-origin distances compared with DMS114 (Fig. 1J–L). Shorter inter-origin distances can result from activation of dormant origins due to oncogene-induced replication stress which slows or stalls replication forks (37). Furthermore, the patterns of bidirectional fork movement were more asymmetric in H524 cells compared with DMS114 (Fig. 1M and N), indicating that higher repstress gene expression associates with replication fork stalling.

Together, we find that the molecular components involved in replication stress response are interconnected. Repstress score captures the coordinate expression of key components of this cascade downstream of checkpoint sensors and kinases with the associated chromatin changes. Even in an unchallenged S-phase, high repstress score cells exhibit more endogenous replication stress and robust activation of DNA damage response (DDR) than low repstress cells. However, they are hypersensitive to exogenous replicative stress likely because further recruitment of replication stress response is less effective. Thus, the repstress gene signature could allow for interrogation of endogenous replication stress and efficiency of the replication stress response in SCLC cell lines.

### Repstress Score Captures Transcriptional Responses to Replication Stress Across Cancer Types

**FIGURE 2:**
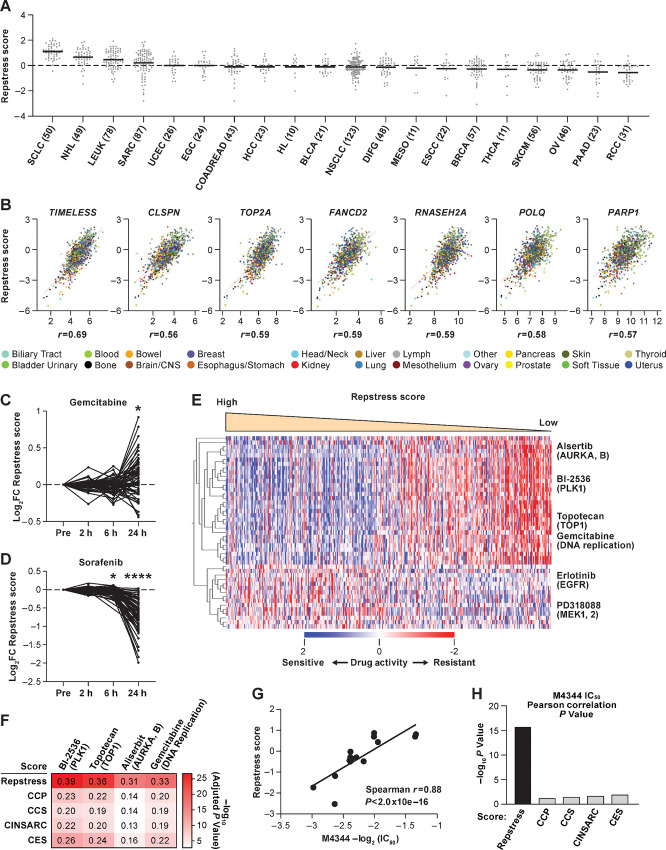
Across cancer cell lines, repstress score profiles replication stress at a functional network level. **A,** Dot plot showing distribution of repstress score across 839 cancer cell lines from 20 cancer types represented in the CCLE. A black bar in each cancer type indicates the mean repstress score within each cancer type. Dashed line indicates zero of Z-normalized repstress score across all of cancer cell lines in CCLE. The numbers with cancer type labels on *x*-axis indicate the numbers of cell lines included. **B,** Across cancers, repstress score correlates with expression of representative genes involved in: (i) increasing replication stress tolerance by protecting replication forks (*TIMELESS*, *CLSPN*), (ii) solving topological problems during replication (*TOP2A*), (iii) facilitating the repair and restart of stalled replication forks (*FANCD2*), (iv) resolving barriers to replication fork progression (*RNASEH2A*), and (v) DNA damage repair factors (*POLQ* and *PARP1*). Correlations were analyzed in CellMiner CDB (16). Spearman correlation coefficients (*r*) are indicated. All of *P* values by Spearman correlation test are <0.0001. Dynamics of normalized repstress score with treatment of gemcitabine (**C**) and sorafenib (**D**) in NCI60 cell lines. Dynamics of gene expression pretreatment and posttreatment are retrieved from The NCI Transcriptional Pharmacodynamics Workbench (42). *, *P* < 0.05; ****, *P* < 0.0001 by Wilcoxon signed-rank test. For detailed method, please refer the Supplementary Materials and Methods. **E,** Heatmap of sensitive or resistant agents in cell lines with high versus low repstress score in the CTRP. Drug activity scores indicate calculated AUC over a 16-point concentration range using an automated, high-throughput workflow fitting concentration–response curves (43). The drug activity scores were retrieved from CellMiner CDB (16) and z score normalized in the heatmap. Cell lines are sorted by repstress score from high (left) to low (right). The heatmap shows 30 mostly sensitive compounds in high repstress score cell lines, and all of sensitive compounds in low repstress score cell lines with FDR of <5%. For detailed methods, please refer Supplementary Materials and Methods. **F,** Heatmap of Pearson correlations between gene signature scores and activities of drugs targeting replication stress. The color in each column indicates log-transformed *P* value of Pearson correlation between annotated gene signature score and drug activity score. The number in each column shows Pearson correlation coefficient between them. CCP, CCS, CINSARC, and CES scores are calculated as reported previously (39, 45–47). **G,** Correlations between half maximal inhibitory concentration (IC_50_) values of M4344 (an ATR-related inhibitor) and repstress score in different cancer type cell lines. The IC_50_ value of M4344 in different cancer type cell lines was examined in a previous report (48). **H,** Comparison of Spearman correlations between M4344 IC_50_ values and scores of repstress and other cell proliferation gene signatures. Each bar represents log-transformed *P* value of Spearman correlation between annotated gene signature and M4344 IC_50_ values. The IC_50_ value of M4344 in different cancer type cell lines is examined in a previous report (48). CCP, CCS, CINSARC, and CES scores are calculated as reported previously (39, 45–47). NHL, non–Hodgkin lymphoma; LEUK, leukemia; SARC, sarcoma; UCEC, uterine endometrioid cancer; EGC, esophagogastric adenocarcinoma; COADREAD, colorectal adenocarcinoma; HCC, hepatocellular carcinoma; HL, Hodgkin lymphoma; BLCA, bladder urothelial carcinoma; NSCLC, non–small cell lung cancer; DIFG, diffuse glioma; MESO, mesothelioma; ESCC, esophageal squamous cell carcinoma; BRCA, breast carcinoma; THCA, thyroid cancer; SKCM, skin melanoma; OV, ovarian cancer; PAAD, pancreatic adenocarcinoma; RCC, renal cell carcinoma; FC, fold change; hr, hour; AURKA, B, aurora kinase A and B; PLK1, polo-like kinase-1; TOP1, topoisomerase I; MAPK1, 2, mitogen-activated protein kinase kinase 1 and 2;CCP, cell-cycle progression; CCS, cell-cycle score; CINSARC, complexity index in sarcomas; CES, Centromere and kinetochore gene expression score.

To determine whether the repstress gene signature was generalizable and able to predict replication stress response signaling in cancers beyond SCLC, we queried RNA-seq and RPPA data from the CCLE of 937 cell lines across 20 cancer types (Fig. 2A; ref. 38). Highest repstress scores were found in SCLC (the number and proportion of SCLC cells with repstress score ≥95% confidence interval of repstress score across all CCLE cell lines: 48/50, 96.0%), hematopoietic malignancies [non–Hodgkin lymphoma (43/49, 87.8%) and leukemia (57/78, 73.1%)], and sarcoma (55/87, 63.2%), consistent with previous reports of these malignancies exhibiting high replication stress phenotype (39, 40). Low repstress scores were observed in renal cell carcinoma (the number and proportion of cells with repstress score <95% confidence interval of repstress score across all CCLE cell lines: 22/31, 71.0%), pancreatic cancer (15/23, 65.2%), ovarian cancer (30/46, 65.2%), melanoma (35/56, 62.5%), and thyroid cancer (6/11, 54.5%). The distribution of repstress score across cancer types was overall similar when DNA repair genes associated with NE were excluded from the signature, with SCLC and hematopoietic malignancies exhibiting the highest scores (Supplementary Fig. S7), suggesting that the high repstress score in SCLC is not confounded by NE, a pathophysiologic characteristic of this cancer.

Similar to SCLC cell lines, the repstress score was positively correlated with expression of key genes involved in increasing replication stress tolerance across cancer types (Fig. 2B). Pairwise correlations recapitulated the correlation of repstress score with expression of DDR mediators, effectors, and heterochromatin, in contrast to sensors and sensor kinases at the mRNA and protein levels (Supplementary Fig. S8).

Genotoxic agents currently used for cancer therapy include many potent inducers of replication stress, such as platinum derivatives, topoisomerase inhibitors, and nucleotide analogs (41). We hypothesized that repstress gene signature may profile drug induced modulation of replication stress in diverse cancers types. To investigate this possibility, we examined repstress score dynamics pretreatment and posttreatment with 15 anticancer agents across a panel of 60 human cancer cell lines of different lineages (42). Cells were exposed to these agents at concentrations below the human peak plasma concentration and the average concentration resulting in 50% cell growth inhibition. In a group of cell lines, we identified similar transcriptional responses to gemcitabine, cisplatin, and topotecan, which resulted in notable induction of repstress gene expression after treatment (Fig. 2C; Supplementary Fig. S9A–C). Topotecan and cisplatin induce replication blocks respectively by generating topoisomerase I−DNA cleavage complexes and platinum–DNA adducts, whereas gemcitabine stalls replication through its integration into DNA and depletion of the deoxyribonucleotide pool. In contrast, treatment with tyrosine kinase inhibitors sorafenib and dasatinib, and the histone deacetylase inhibitor vorinostat resulted in uniformly decreased repstress gene expression (Fig. 2D; Supplementary Fig. S9A, S9D, and S9E).

Together, repstress gene signature stratifies cancer cell lines across tumor types based on their adaptability to replication stress and profiles transcriptional responses to drug-induced modulation of replication stress. Molecular features that contribute to the replication stress phenotype including drug responses across cancer cell line databases may be explored at this web-based resource: https://discover.nci.nih.gov/cellminercdb/ (15, 16).

### Repstress Score Predicts Sensitivity to Replication Stress–Targeted Therapies Including Novel ATR Inhibitors

Cancers with heightened replication stress response may be particularly vulnerable to drugs that target this dependency. We investigated whether the repstress score predicts drug sensitivity using 481 anticancer drugs across 823 cell lines of the CTRP (43). Drug sensitivities were compared between cell lines defined by the lowest (<25th) and highest (≥75th) repstress score percentiles. With FDR of 5%, 280 compounds were identified as significantly more or less active in repstress-high compared with repstress-low cell lines (Supplementary Fig. S10A). High repstress score cells were more sensitive to inhibitors of polo-like kinase-1 (BI-2536: *P*_adj_ = 2.4 × 10^−28^), topoisomerase I (topotecan: *P*_adj_ = 1.1 × 10^−21^), aurora kinase A and B (alisertib: *P*_adj_ = 2.0 × 10^−20^), and regulators of cell-cycle progression and DNA replication (gemcitabine: *P*_adj_ = 9.4 × 10^−17^; Fig. 2E; Supplementary Fig. S10). In contrast, low repstress score cells were more sensitive to compounds targeting pathways such as mitogen-activated protein kinase (MAPK) and EGFR (Fig. 2E; Supplementary Fig. S10A). This observation is consistent with a recent study in isogenic cell lines which reported MAPK signaling dependence in replication stress response defective cells (44). Repstress score exhibited a higher positive correlation with response to agents that induce replication stress, including alisertib, BI-2536, topotecan, and gemcitabine, than the currently available cell-cycle proliferation genes (refs. 39, 45–47; Fig. 2F, Supplementary Fig. S11).

Because of the critical functions of ATR in protecting cells under replication stress, small-molecule ATR inhibitors are being explored as cancer therapeutic agents to selectively kill cancer cells under replication stress (9). A reliable method to measure replication stress levels could in principle enable patient stratification for ATR inhibitor therapies. We examined whether the repstress signature predicted sensitivity to ATR inhibitors (48). Across 16 cancer cell lines from different histologies, cells with high repstress score showed higher sensitivity to ATR inhibitor M4344 than cells with low repstress score (Spearman *r* = 0.88, *P* < 2.0 × 10^−16^; Fig. 2G). Repstress score better predicted ATR inhibitor response than the previously described signatures of replication stress and proliferative gene expression signatures (Fig. 2H; Supplementary Fig. S12; refs. 39, 45–47).

### Repstress Score Defines Subsets of Cancers Characterized by Genomic Instability, Immune Evasion, and Poor Prognosis Across Tumor Types

**FIGURE 3:**
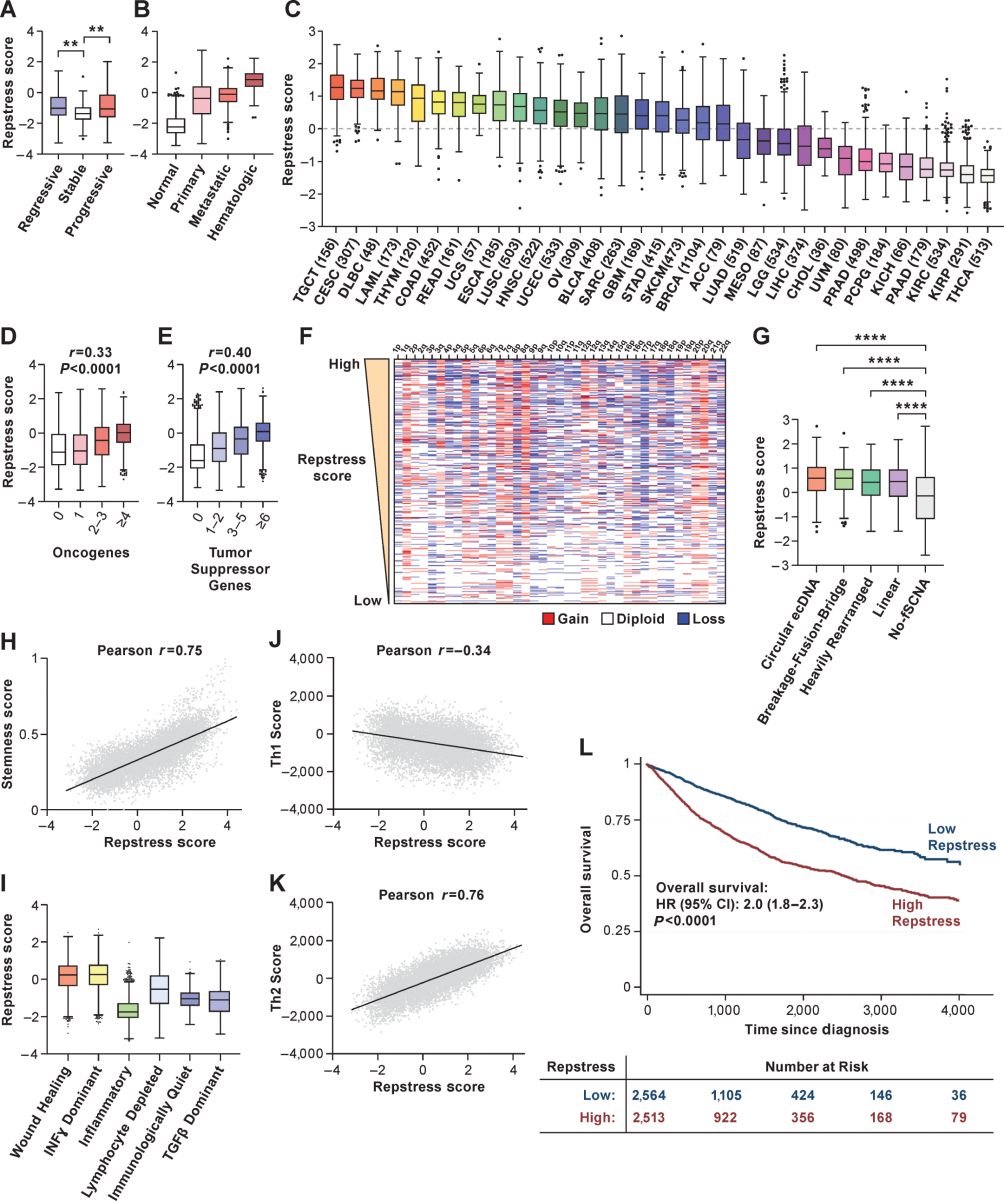
Across cancer types, repstress score defines cancers characterized by genomic instability, immune evasion, and poor prognosis. **A,** Comparison of repstress score among bronchial premalignant lesions which regressed to normal tissue (regressive), did not change the premalignant histology (stable), and progressed to invasive malignancy (progressive) after biopsy. Gene expression data are obtained from a previous report (50). **, *P* < 0.01 by one-way ANOVA followed by Tukey multiple comparison test. **B,** Comparison of repstress score among TCGA normal tissue, primary and metastatic epithelial cancers, and hematopoietic malignancies *P* < 0.0001 by comparing repstress scores in normal tissues versus primary and metastatic epithelial cancers, and hematologic malignancies; and comparing those in hematologic malignancies versus primary cancer and metastatic cancers; whereas *P* > 0.05 comparing those in primary and metastatic epithelial cancers. *P* values are analyzed by one-way ANOVA followed by Tukey multiple comparison test. **C,** Distribution of repstress scores across 33 cancer types in TCGA The number in the *x*-axis label indicates the number of tumors included in each cancer type. A dash line indicates zero of Z-normalized repstress score across all of tumors in TCGA. Pan-cancer analysis showing the relationship between repstress score with the number of mutated oncogenes (**D**) and tumor suppressor genes (**E**; ref. 54). Spearman correlation coefficient (*r*) and *P* values are indicated on top of each panel. Hypermutated tumors (i.e., mutational burden of ≥ 50 mutations per megabase) are excluded. **F,** Copy-number alteration heatmap sorted by high (top) to low (bottom) repstress score. Chromosome with copy-number deletion or gain are indicated with blue and red, respectively. Copy-number alteration data in TCGA tumors are retrieved from a previous report (81). **G,** Comparison of repstress scores among tumors with amplicons of circular ecDNA, breakage-fusion-bridge, heavily rearranged, linear, and no focal somatic copy-number amplification Annotations of amplification for each tumor in TCGA are reported previously (55). ****, *P* < 0.0001 by one-way ANOVA followed by Tukey multiple comparison test. **H,** Correlation between cancer stemness score and repstress score. Cancer stemness score is derived by integrative transcriptome- and methylation-based analysis (57). The *P* value of Pearson correlation is <0.0001. **I,** Comparison of repstress score across six distinct TCGA immune subtypes, derived by gene signature–based clustering approach. Immune subtypes are described previously (58). *P* < 0.0001 by comparing repstress score in wound healing group versus the others; IFNγ dominant group versus the others; and inflammatory versus the others, respectively. *P* values are analyzed by one-way ANOVA followed by Tukey multiple comparison test. Correlations between Th1 (**J**) and Th2 (**K**) scores, and repstress score across cancer types Th1 and Th2 scores are available in a previous report (58). The *P* values of Pearson correlation are <0.0001 in **J** and **K**. **L,** OS in patients with cancer with high versus low repstress score. High versus low repstress scores are defined as patients whose cancers have repstress score ≥75th or <25th percentiles across TCGA tumors. *P* value is derived from the log-rank test. TCGA: The Cancer Genome Atlas; fSCNA: focal somatic copy-number alteration; CI, confidence interval. Abbreviations for cancer types in TCGA are available from https://gdc.cancer.gov/resources-tcga-users/tcga-code-tables/tcga-study-abbreviations.

Replication stress is a driver for cancer progression and is linked to genomic instability in precancerous lesions and cancers (7). In precancerous lesions, the replication stress response provides a barrier to delay or prevent tumorigenesis (6, 8, 49). Using repstress score, we assessed replication stress along the continuum of cancer development (50). Repstress scores were higher in bronchial precancerous lesions which eventually regressed and those that progressed to become cancers, compared with lesions that maintained stable precancerous characteristics (Fig. 3A), supporting the dual roles of replication stress in promoting genomic instability, and in slowing down cell proliferation and activating anticancer barriers (8).

To explore the replication stress response profiles of cancers, we analyzed over 10,000 tumors of 33 cancer types from TCGA. As with cell lines, expression of genes required for survival of replication stress and DNA damage repair (*TIMELESS, CLSPN, TOP2A*, *FANCD2*, *RNASEH2A*, *POLQ*, and *PARP1*) positively correlated with repstress scores (Supplementary Fig. S13A–G). These associations were also maintained at the protein level across tumor types; expression of proteins that most highly correlated with repstress score included CYCLINB1, CYCLINE1, CHK2, 4EBP1, phosphorylated CDK1 and PCNA (Supplementary Fig. S13H). We next assessed repstress scores across normal tissue, localized, and metastatic cancers. Normal tissue had the lowest repstress score compared with cancers, and hematologic malignancies had higher repstress score than epithelial cancers (Fig. 3B).

We observed large variance in repstress scores across cancer types, implying significant differences in replication stress response proficiency among different cancers (Fig. 3C). High repstress gene expression was observed in testicular germ cell tumors (TCGT, the number and proportion of TCGT with repstress scores ≥95% confidence interval of repstress score across TCGA: 148/156, 94.9%), cervical squamous cell carcinoma (302/307, 98.4%), and hematologic malignancies (diffuse large B-cell lymphoma: 46/48, 95.8%; and acute myeloid leukemia: 161/173, 93.1%). In general, tumors with high repstress scores were highly proliferative tumors typically treated with DNA-damaging therapies such as platinum and topoisomerase inhibitors. A notable exception was thymoma which had high repstress scores (THYM: 96/120: 80.0%) despite a relatively indolent growth pattern. This may be explained by the prominent role of E2F2 in promoting unscheduled cell division and oncogenic transformation of thymic epithelial cells (51). Cancer types with lower repstress scores included thyroid cancers (THCA: the number and proportion of THCA with repstress scores <95% confidence interval of repstress score across TCGA: 513/513, 100%), kidney cancers [renal papillary cell carcinoma (KIRP): 284/291, 97.6%; renal clear cell carcinoma (KIRC): 521/534, 97.6%; kidney chromophobe (KICH): 63/66, 95.5%], and pancreatic adenocarcinoma (PAAD: 172/179, 96.1%). The distribution of repstress score across cancers was overall similar even after excluding the seven genes associated with NE differentiation (Supplementary Fig. S14).

Because replication stress is driven by activation of oncogenes and absence of tumor suppressor genes (52), we examined the association between repstress score and mutations or copy-number states in these genes. Tumors with mutated oncogenes (Fig. 3D) and tumor suppressor genes (Fig. 3E) had higher repstress scores compared with tumors with no mutations affecting these genes. In most cancer types, repstress score was significantly higher in tumors harboring mutations in DNA repair and cell cycle–related genes (Supplementary Fig. S15A), suggesting deregulation of these pathways underlying increased replication stress. Tumors with *TP53* or *RB1* mutations had significantly higher repstress score compared with those without (Supplementary Fig. S15B and S15C) and a loss of Rb1 function score (53) positively correlated with repstress score (Supplementary Fig. S15D). Notably, there was no association between repstress score and the number of point mutations (Supplementary Fig. S15E). In contrast, somatic copy-number alterations (54) at chromosome, arm, and focal levels (Fig. 3F; Supplementary Fig. S15F) and whole-genome doubling (Supplementary Fig. S15G) were positively correlated with repstress score. Extrachromosomal DNA (ecDNA) amplification has recently been reported to promote aneuploidy and genomic instability (55). Tumors with ecDNA amplification had higher repstress scores compared with those without (Fig. 3G), with increasing number of ecDNA amplicons associated with higher repstress scores (Supplementary Fig. S16). Consistent with cancer stem cells displaying robust replication stress response to prevent the accumulation of genetic lesions (56), a cancer stemness gene signature score (57) positively correlated with repstress score (Fig. 3H).

Next, we examined repstress score among previously defined cancer immune subtypes (58). The wound healing and IFNγ dominant subtypes had higher repstress scores compared with the other immune subtypes, including notably the inflammatory subtype which had lower repstress scores (Fig. 3I). The association of wound healing and repstress score (Pearson *r* = 0.81, *P* < 0.0001; Supplementary Fig. S17A; ref. 58), consistently observed across nearly all cancer types (Supplementary Fig. S17B), is supported by previous work showing the similarities in cellular responses to cancer progression and wound healing (59). Th cells play a key role in the adaptive immune system by coordinating effector functions leading to destructive responses, including pathogen clearance and autoimmunity. A proinflammatory Th1 subtype response score was negatively correlated with repstress score (Pearson *r* = −0.34, *P* < 0.0001), whereas immunosuppressive Th2 subtype response score correlated positively (Pearson *r* = 0.76, *P* < 0.0001; Fig. 3J and K). Accordingly, high repstress score was associated with poor survival in an independent cohort of melanoma patients treated with immune checkpoint inhibitor nivolumab (ref. 60; Supplementary Fig. S18).

Finally, we analyzed the impact of repstress score on patient outcomes. Patients with high repstress tumors had poorer OS compared with patients with low repstress tumors [HR (95% confidence interval): 2.0 (1.8–2.3), *P* < 0.0001 by log-rank test; Fig. 3L]. Multivariate Cox regression analysis revealed that the repstress score independently contributed to poor survival after adjusting known variables associated with survival including age at diagnosis, sex, pathologic/clinical stage, and cancer type (Supplementary Table S2; Supplementary Fig. S19). Together, these analyses functionally link replication stress and its cellular response as measured by the repstress score with oncogene alterations, tumor aneuploidy, ecDNA amplification, cancer stemness, immunosuppressive T-cell responses, and inferior survival across cancers.

### Repstress Score Defines Distinct Molecular Subtypes Within Cancer Types

**FIGURE 4:**
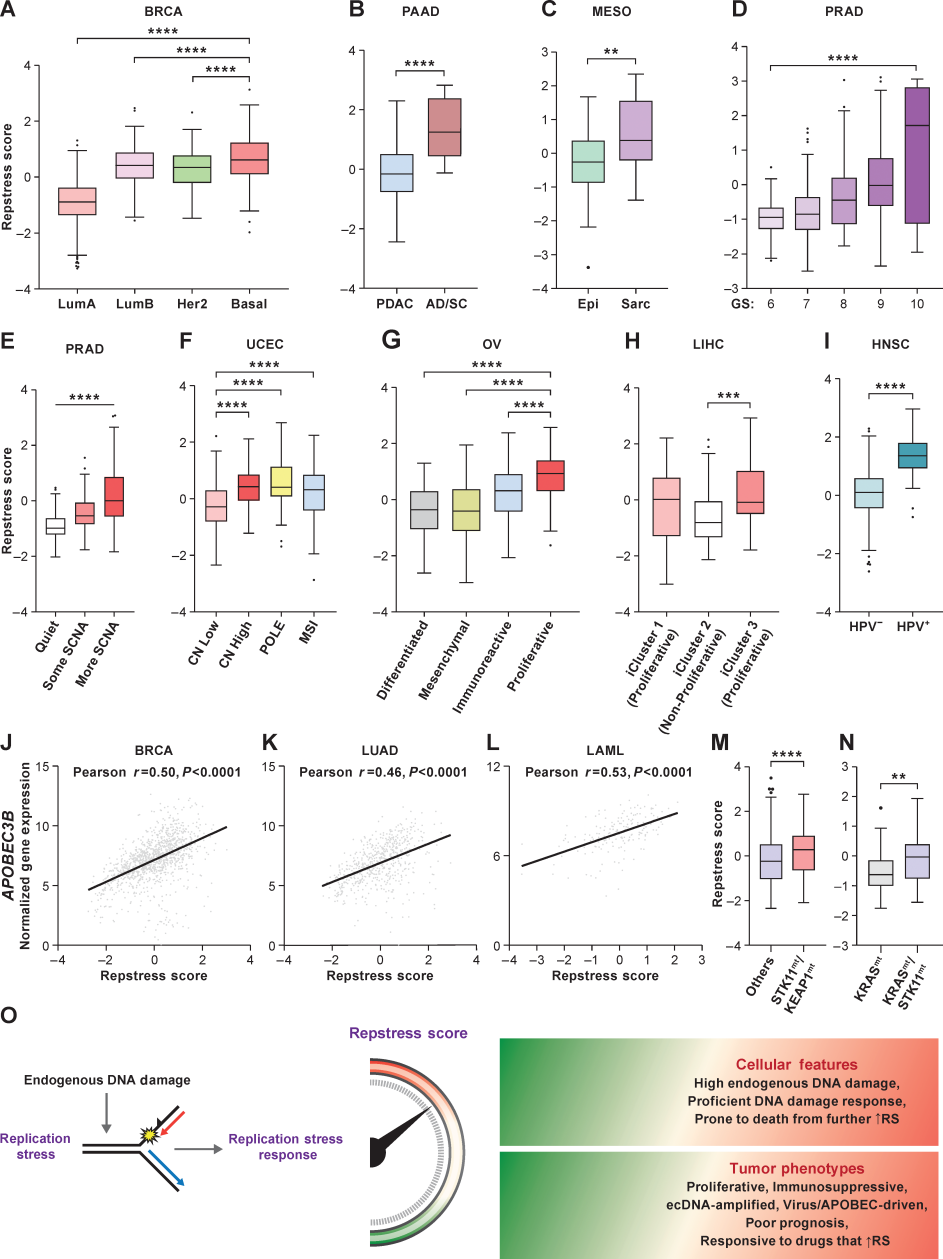
Resptress score identifies distinct molecular subtypes among various cancer types. **A,** Repstress scores among different breast cancer molecular subtypes. ****, *P* < 0.0001 by one-way ANOVA followed by Tukey multiple comparison test. **B,** Repstress scores in pancreatic cancers with adenocarcinoma (PDAC) versus adenosquamous (AD/SC) histology. ****, *P* < 0.0001 by Mann–Whitney *U* test. **C**, Repstress scores in malignant mesothelioma with epithelioid (Epi) versus sarcomatoid or mixed epithelioid and sarcomatoid (Sarc) histology. **, *P* < 0.01 by Mann–Whitney *U* test. **D**, Repstress scores among prostate cancers with different Gleason scores. ****, *P* < 0.0001 by linear trend test from left to right. **E,** Repstress scores and somatic copy-number alterations (SCNA) of TCGA prostate cancers SCNA subtype are defined by copy number–based clustering in a previous report (64). ****, *P* < 0.0001 by linear trend test from left to right. **F,** Repstress scores among uterine corpus endometrial carcinomas with different SCNA subtypes. SCNA subtypes are defined by copy number–based clustering in a previous report (65). ****, *P* < 0.0001 by one-way ANOVA followed by Tukey multiple comparison test. **G,** Repstress scores among transcriptomic subtypes in ovarian carcinoma The molecular subtypes are defined on the basis of transcriptome-based clustering in a previous report (66). ****, *P* < 0.0001 by one-way ANOVA followed by Tukey multiple comparison test. **H,** Repstress scores among genomic subtypes in hepatocellular carcinoma. The molecular subtypes (iCluster) are defined on the basis of an integrative analysis of DNA copy number, DNA methylation, mRNA expression, miRNA expression, and RPPA in a previous report (67). ***, *P* < 0.001 by one-way ANOVA followed by Tukey multiple comparison test. **I,** Repstress scores between patients with HPV-null (HPV−) and HPV-driven (HPV+) head and neck cancers. ****, *P* < 0.0001 by unpaired Student *t* test. Correlations between gene expression of *APOBEC3B* and repstress score in breast cancer (**J**), lung adenocarcinoma (**K**), and acute myeloid leukemia (**L**). Repstress score comparison between tumors with *KEAP1*/*STK11* coalterations compared with those without (**M**), and tumors with *KRAS*/*STK11* coalterations compared with *KRAS* single-altered tumors (**N**) in lung adenocarcinoma. Gene alterations or copy-number deletion (either heterozygous or homozygous) are considered as genetically alteration in *KRAS, KEAP1,* and *STK11*. Lung adenocarcinoma with *KRAS*/*TP53* or *KRAS*/*CDKN2A* comutations are excluded from the analysis in **N** given a previous study reporting that non–small cell lung cancer with these comutations is different subtype from *KRAS/STK11* comutated subtype (71, 72). ****, *P* < 0.0001; **, *P* < 0.01 by Mann–Whitney *U* test. **O,** A schema of repstress gene signature characterizing replication stress and its response. TCGA, The Cancer Genomic Atlas; LumA, luminal A; LumB, luminal B; PDAC, pancreatic adenocarcinoma; AD/SC, adenosquamous; Epi, epithelioid; Sarc, sarcomatoid; GS, Gleason score; SCNA, somatic copy-number alteration; CN, copy number; POLE, DNA polymerase epsilon, catalytic subunit; MSI, microsatellite instable; HPV, human papillomavirus; APOBEC, apolipoprotein B mRNA editing enzyme, catalytic polypeptide-like; STK11, Serine/threonine kinase 11; KEAP1, Kelch-like ECH-associated protein 1; RS, replication stress. Abbreviations for cancer types in TCGA are available from https://gdc.cancer.gov/resources-tcga-users/tcga-code-tables/tcga-study-abbreviations.

Given the wide range of repstress scores in individual cancers (Fig. 3C), we hypothesized that the repstress score can identify distinct molecular subtypes within cancer types. Among breast cancers, the basal subtype, characterized by expression of markers such as cytokeratins 5 and 6 (61), had significantly higher repstress score compared with the luminal A, luminal B, and HER2-enriched subtypes (Fig. 4A). Triple-negative breast cancers, which share similarities to the basal subtype, were also characterized by higher repstress score gene expression than tumors that expressed estrogen, progesterone, or HER2 receptors (Supplementary Fig. S20A). Pancreatic cancers with transcriptionally defined basal characteristics and squamous features on histology harbored higher repstress score than those without these features in TCGA and an independent cohort (Fig. 4B; Supplementary Fig. S20B–S20F; ref. 62). Malignant mesothelioma with sarcomatoid histology, defined by infiltrative spindle or mesenchymal appearing cells and poor prognosis, were characterized by higher repstress score than epithelioid mesothelioma (Fig. 4C). Among prostate cancers, repstress score showed a positive correlation with Gleason score (Fig. 4D), an indicator of prostate cancer differentiation, with the highest Gleason score associated with the most poorly differentiated and aggressive subtype (63). In addition, prostate cancers with higher copy-number alterations (64) had higher repstress scores compared with those with less frequent copy-number alterations (Fig. 4E). Similarly, uterine corpus endometrial carcinoma with genomic instability defined by high copy-number alterations, *POLE* mutations, and microsatellite instability (65) had higher repstress score compared with low copy-number altered tumors (Fig. 4F). Repstress score also identified a proliferative subtype of ovarian cancer (ref. 66; Fig. 4G), and aggressive subtypes of hepatocellular carcinoma (iCluster 3; ref. 67) with higher degree of chromosomal instability and *TP53* mutations (Fig. 4H).

Given recent studies linking oncoviruses with genomic instability and replication stress (68), we examined repstress score in oncovirus-derived cancers. Human papilloma virus (HPV)-associated head and neck cancers had significantly higher repstress scores compared with non–HPV-associated cancers (Fig. 4I). A similar trend was also observed in cervical cancer, another HPV-related cancer (Supplementary Fig. S20G). Replication stress exposes tracts of ssDNA that form substrates for APOBEC3-deaminase–mediated mutagenesis (69). Accordingly, repstress score positively correlated with *APOBEC3B* expression in breast cancer, lung adenocarcinoma, and acute myeloid leukemia, malignancies wherein *APOBEC3B* is upregulated and plays a key role in mutagenesis (ref. 70; Fig. 4J–L). *STK11* and *KEAP1* co-mutated lung adenocarcinoma, which are associated with aggressive tumor growth and immunotherapy resistance (71), had higher repstress scores compared with lung adenocarcinoma without concomitant loss of these genes (Fig. 4M). Among *KRAS*-mutant lung adenocarcinoma, a particularly aggressive subset with *STK11* comutations (72) had higher repstress scores compared with tumors without comutations (Fig. 4N). Non–small cell lung cancer cell lines with *KRAS*/*STK11* comutations were more sensitive to a CHK1/2 inhibitor than cell lines without *STK11* comutations (Supplementary Fig. S21). Together, our analysis brings to light the dependence of certain tumor types and subtypes of tumors on replication stress response, potentially representing important therapeutic opportunities.

## Discussion

DNA replication is a tightly regulated process. Replication stress and DNA damage ensue when these regulatory mechanisms fail. Causes of replication stress are diverse. Even single oncogenes can induce replication stress by different mechanisms depending on the context (73). In fact, the causes of replicative stress might be quite dynamic during tumorigenesis. Independent of the causes of replication stress, cells have evolved a complex mechanism which ensures that the genome is accurately duplicated in each cell cycle. Despite its critical role in tumorigenesis and emerging importance as a potential therapeutic target, replication stress and its phenotypic characteristics have not been explored in high-throughput sequencing studies of human cancers. Many available studies examining replication stress to date have focused on individual tumor types, for example in ovarian cancer (74), pancreatic cancer (75, 76), or selected features that drive replication stress, for example overexpression of oncogenes (via overexpression of *CDC25A*, *CCNE1* or *MYC*; ref. 77) or replication stress response defects (via depletion of ATR, ATM, CHEK1, or CHEK2; ref. 44). Here we describe a gene expression signature, capturing broad measures of replication stress–related gene expression using an approach compatible with formalin-fixed paraffin-embedded clinical samples, allowing interrogation of replication stress at a functional network level across cancers, independent of the underlying mechanisms. The global view of replication stress provided by the repstress signature reveals heightened genomic instability, immune evasion, and poor survival in subsets of tumors across lineages, and enabled identification of cancer subtypes that may be more vulnerable to replication and replication stress response inhibitors including the novel ATR inhibitors (Fig. 4O; Supplementary Fig. S1).

Repstress score provides a framework to investigate the link between replication stress and its functional consequences. Our analyses implicate copy-number alterations rather than base-pair mutations as a key consequence of genomic instability linked to DNA replication stress. These results support the oncogene-induced DNA replication stress model for cancer development wherein chromosomal instability in sporadic cancers results from oncogene-induced collapse of DNA replication forks, which in turn leads to DNA double-strand breaks and genomic instability (78). Another consequence of replication stress is abnormal chromosome segregation which may result in formation of micronuclei (79) and nonchromosomal DNA elements (55). Indeed, we find a positive correlation between repstress gene expression and ecDNA amplification, suggesting that oncogene-induced replication, abnormal chromosome segregation, and chromosome instability may be driving ecDNA formation.

Repstress gene signature reveals the dynamic nature of the replication stress response during tumorigenesis and following drug treatment. Bronchial precancerous lesions that eventually regress and those that progress to become cancers are characterized by high repstress score compared with lesions that maintain stable precancerous characteristics. These results are consistent with the fundamental role of replication stress response in early stages of cancer development maintaining genomic integrity and preventing tumorigenesis (6, 8) while generating DNA damage and contributing to rapid evolution and genetic heterogeneity in established cancers (52). Whether these insights could enable the currently sparse toolset to identify and treat premalignant lesions at risk for progression to cancer needs further study (80). Modulation of repstress score following treatment suggests the utility of the signature to profile to study agents in terms of their impact on replication stress.

Repstress score provides insights into tumor phenotypes associated with high replication stress. Across multiple datasets, repstress score was an independent predictor of poor survival after adjusting known variables associated with survival. Notably, we find substantial enrichment of TCGA wound healing and IFNγ dominant phenotypes among high repstress tumors. The dominant anti-inflammatory Th2 response and rapid tumor growth that preclude immune control may explain the notably less favorable outcomes in high repstress score tumors despite a substantial immune component. It is also likely that these tumors have already been remodeled by the existing robust Th1 infiltrate and have escaped immune recognition. Furthermore, the repstress score enabled delineation of several prognostically relevant subtypes within diverse cancer types, including high Gleason score prostate cancer, basal subtype of breast cancer, sarcomatoid mesothelioma, proliferative subtypes of ovarian cancer and hepatocellular carcinoma, and pancreatic cancer with squamous differentiation.

Additional studies are warranted to define clinically relevant and tumor-type specific repstress score thresholds, but it is notable, and probably the singular strength of the study, that repstress gene signature stratifies tumors across and within cancer types beyond SCLC based on the likelihood of drug response and prognosis. The generalizability of repstress score beyond SCLC suggests that while the causes of replication stress are varied, the replication stress response pathways are conserved across cancers, and thus may represent a shared therapeutic vulnerability. Upregulation of cell-cycle genes is a common denominator between highly proliferative cells and cells under high replication stress, and further studies are needed to understand the contribution of individual repstress genes to these characteristics. It is notable that repstress signature better predicted response to ATR inhibitors than previously described gene signatures of proliferation (39, 45–47), suggesting that repstress signature captures transcriptional changes of replication stress in addition to proliferation. In conclusion, gene expression profiling–based assessment of replication stress using the repstress signature represents a powerful approach to dissect the replication stress response. We anticipate the repstress score to have therapeutic implications, enabling stratification of patients for therapies that modulate replication stress.

## Supplementary Material

Supplementary Data S1 Supplementary Data
