# Supplementary material for: Replication Stress Defines Distinct Molecular Subtypes Across Cancers
Source: Cancer Res Commun. 2022 Jun 24;2(6):503–17. doi: 10.1158/2767-9764.CRC-22-0168 (PMC9648410; doi:10.1158/2767-9764.CRC-22-0168)
Supplement: Supplementary Data S1 — Supplementary Data [file crc-22-0168-s01.docx]

**Replication stress defines distinct molecular subtypes across cancers**

Takahashi N et al.

Supplemental Materials

**Supplementary Text**

Data acquisition

We obtained p-Chk1 expression data (Chk1_pS345) in corresponding small cell lung cancer (SCLC) cell lines from the Cancer Cell Line Encyclopedia (CCLE) (1). Proteome expression data in CCLE cell lines were retrieved form a previous report (2). RNA sequencing data and outcomes of lung precancerous lesions were obtained from Gene Expression Omnibus under accessions code GSE111091 as reported in a previous study (3). Tumor mutational burden data in corresponding samples in the Cancer Genome Atlas (TCGA) were obtained from National Cancer Institute Genomic Data Commons platform (4). Status of human papilloma virus (HPV) infection in head and neck cancers and cervical cancers in TCGA were retrieved from FireBrowse database (5). Data of extrachromosomal DNA (ecDNA) amplifications in TCGA tumors were retrieved from a previous report (6). RNA sequencing data and overall survival in patients with metastatic melanoma treated with nivolumab were obtained from Gene Expression Omnibus under accessions code GSE91061 as reported in a previous study (7). RNA sequencing data for pancreatic cancer autopsy samples as well as histology and molecular subtype annotations for TCGA pancreatic cancer samples were downloaded from a previous report (8).

Repstress score dynamics with cancer drugs

Gene expressions at pre-treatment, 2, 6, and 24 hours after treatment of 15 compounds as well as their concentration causing 50% cell growth inhibition (GI50) were obtained from The NCI Transcriptional Pharmacodynamics Workbench (9) and repstress score at each timepoint was calculated as described in Materials and Methods. Concerning that the repstress score strongly correlated with cell proliferation (**Fig. S8A, B**), reptress score at each time point was respectively divided by GI50 of each drug to normalize the contribution of decreasing proliferation due to their cytotoxicity.

Drug sensitivity and repstress score in Cancer Therapeutics Response Portal (CTRP)

Drug activity scores indicate calculated area under the curve over a 16-point concentration range using an automated, high-throughput workflow fitting concentration-response curves (10). Within CTRP, we first excluded cell lines or compounds whose drug activity scores were not available in ≥ 50% of all of compounds or cell lines, respectively. High or low repstress score was defined as repstress score of ≥ 75th or < 25th percentiles across the cell lines, respectively. Drug activity scores of each compound were statistically compared by unpaired Student t test between cell lines with high vs. low repstress score followed by adjustment of multiple testing by Benjamini-Hochberg test. Adjusted P value < 0.05 was considered as significantly sensitive or resistant compounds.

**Supplementary Figures and Tables**

**
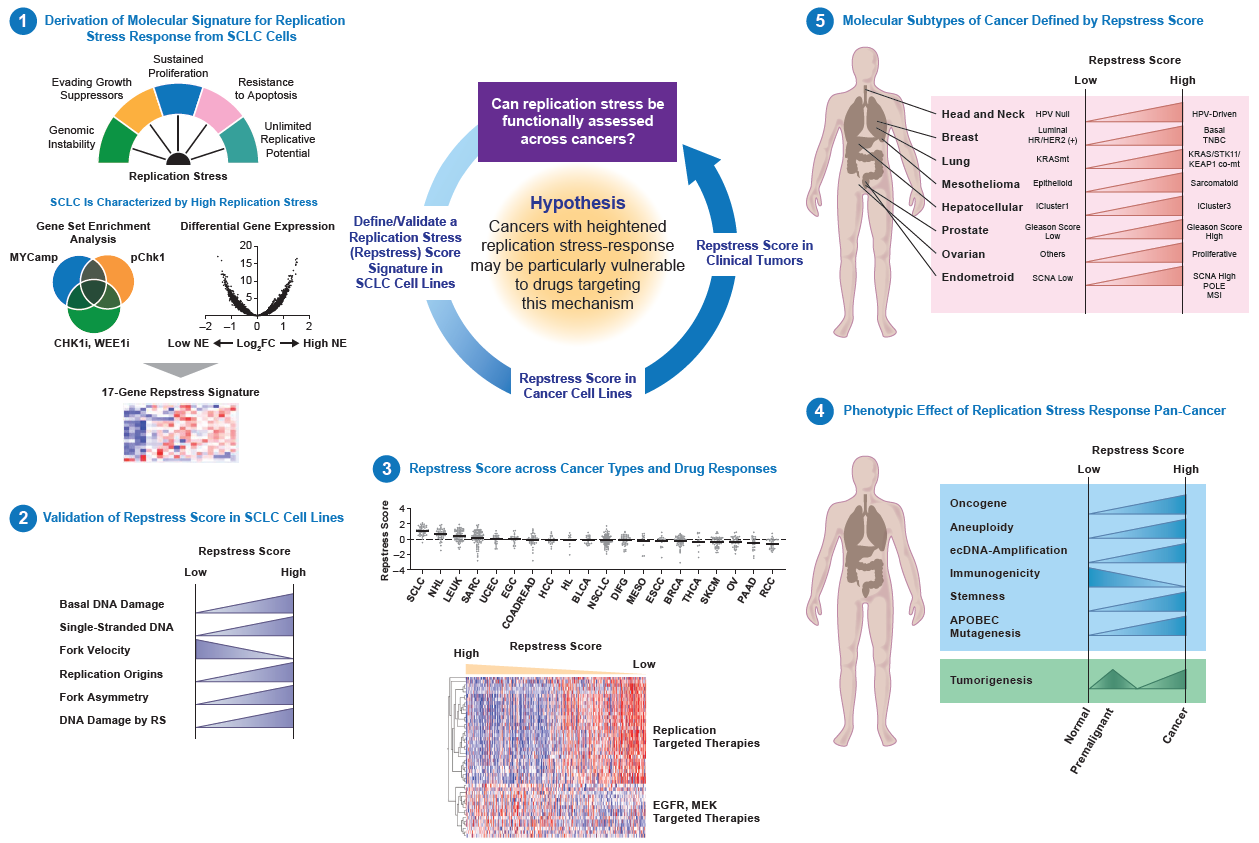
**

**Fig. S1.**Schema of repstress gene signature development and validation

**
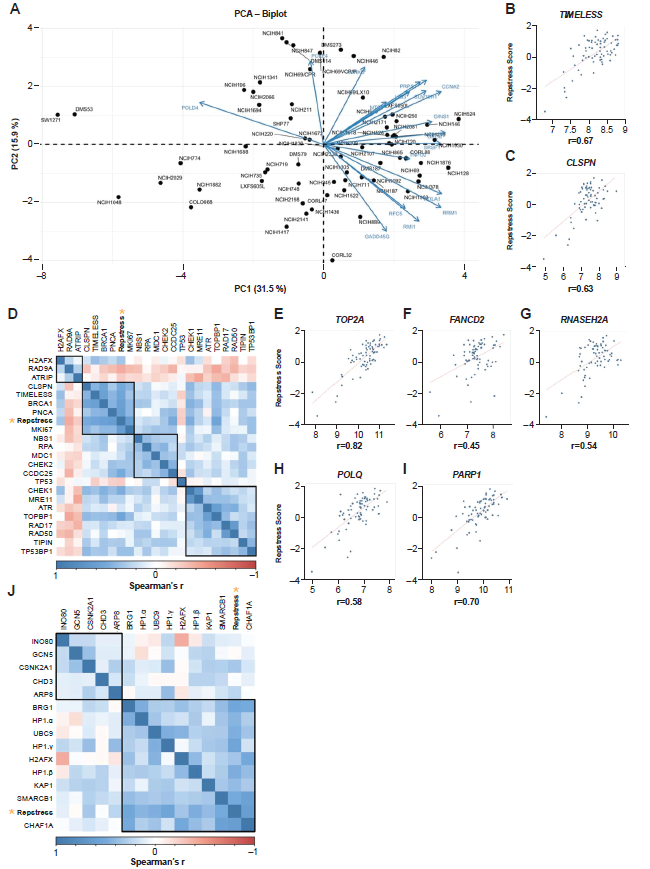

Fig. S2.**Development and validation of repstress gene signature in NCI-DTP small cell lung cancer (SCLC) cell lines
(**A**), Principal component analysis (PCA) of repstress gene set expression data to define weights for each gene, based on the PC1-associated weighting
Values in parenthesis indicate the percent of explained variance.
(**B**), (**C**), Across NCI-DTP SCLC cell lines, repstress score correlates with expression of representative genes involved in increasing replication stress tolerance by protecting replication forks (*TIMELESS* [B], *CLSPN* [C]). Spearman’s correlation coefficients (r) are indicated at the bottom of each scatter plot. P values of Spearman’s correlations in panel (B) and (C) are < 0.0001.
(**D**), Pairwise correlations of repstress score and gene expressions in replication stress-response pathways and proliferation markers in NCI-DTP SCLC cell lines
Genes are clustered by Euclidean distance, using the complete-linkage clustering method, are indicated with squares with black lines. Yellow stars highlight repstress score.
(**E**) to (**I**), Across NCI-DTP SCLC cell lines, repstress score correlates with expression of representative genes involved in: (i) solving topological problems during replication (*TOP2A* [E]), (ii) facilitating the repair and restart of stalled replication forks (*FANCD2* [F]), (iii) resolving barriers to replication fork progression (*RNASEH2A* [G]), and (iv) DNA damage repair (*POLQ* [H], *PARP1* [I]). Spearman’s correlation coefficients (r) are indicated at the bottom of each scatter plot. P values of Spearman’s correlations in panel (E) to (I) are < 0.0001.
(**J**), Pairwise correlations between repstress score and expressions of genes in chromatin modulators in NCI-DTP SCLC cell lines
Genes are clustered by Euclidean distance, using the complete-linkage clustering method, are indicated with squares with black lines. Yellow stars highlight repstress score.
Abbreviations: PC: principal component; PCA: principal component analysis; SCLC: small cell lung cancer.

**
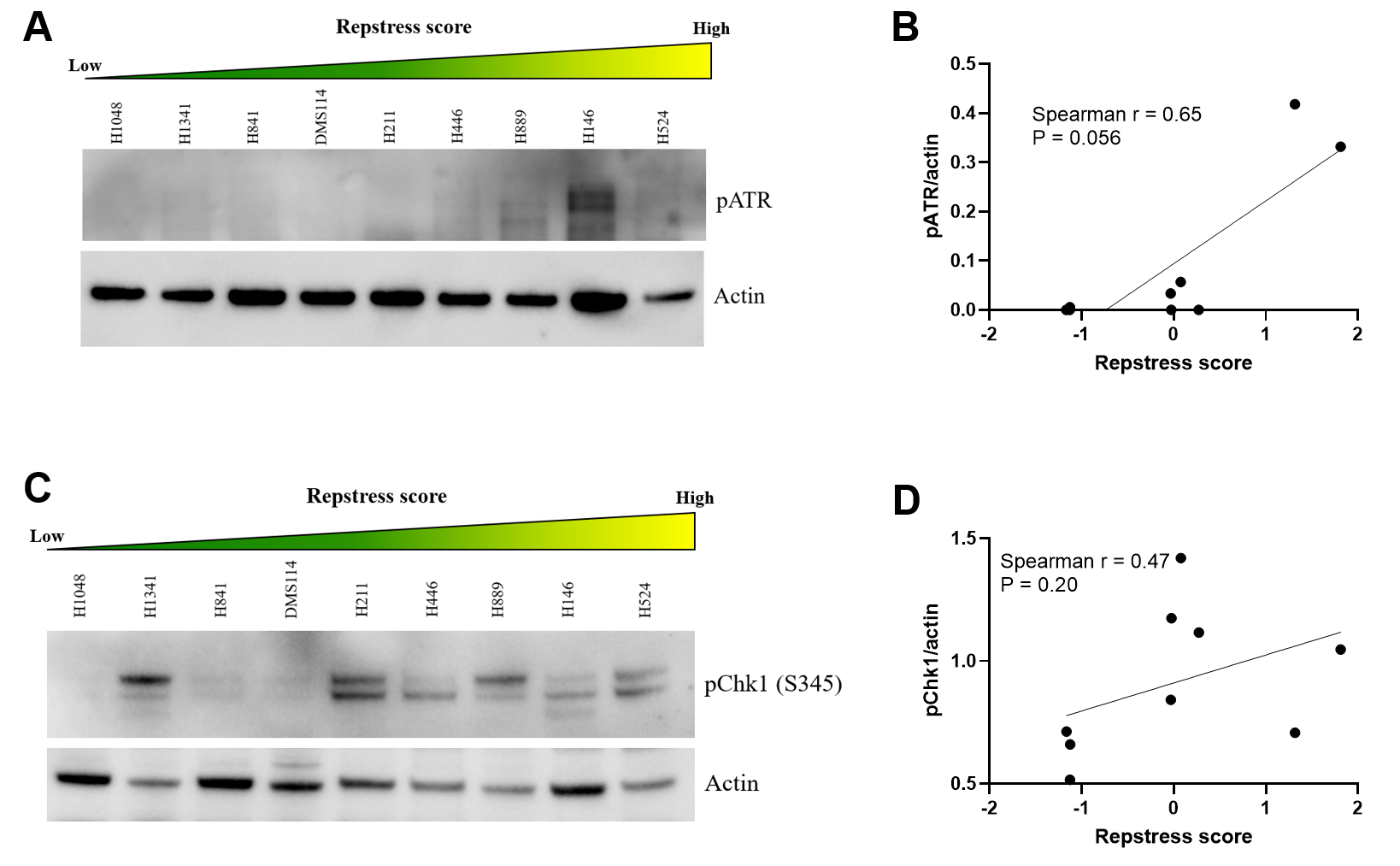
**

**Fig. S3.**Western blots and correlations of pATR (A, B) and pChk1 signals (C, D) with repstress score in small cell lung cancer (SCLC) cell lines
SCLC cell lines are ordered from low to high repstress score (range: -1.9 – 1.5) from left to right in western blot panels (A, C).
Abbreviations: pATR: phosphorylated ataxia telangiectasia mutated- and Rad3-related; pChk1: phosphorylated checkpoint kinase 1.

**
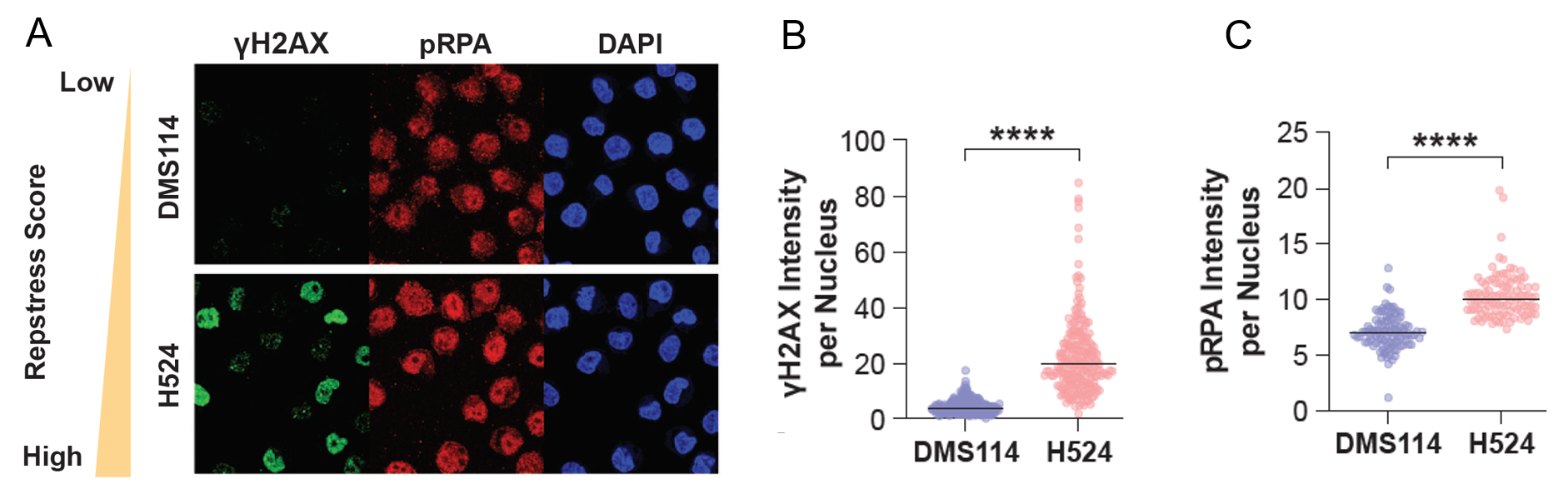
**

**Fig. S4.**

Confocal immunofluorescence microscopy images (A) and quantifications (B, C) of small cell lung cancer (SCLC) cell lines with low (DMS114) and high (H524) repstress score stained for γH2AX, pRPA, and DAPI

Mean γH2AX (B) and pRPA (C) signal intensity in each cell line are indicated with black lines. ****: P < 0.0001 by unpaired Student t test.

Abbreviations: SCLC: small cell lung cancer; pRPA: phosphorylated Replication protein A; DAPI: 4′,6-diamidino-2-phenylindole.

**
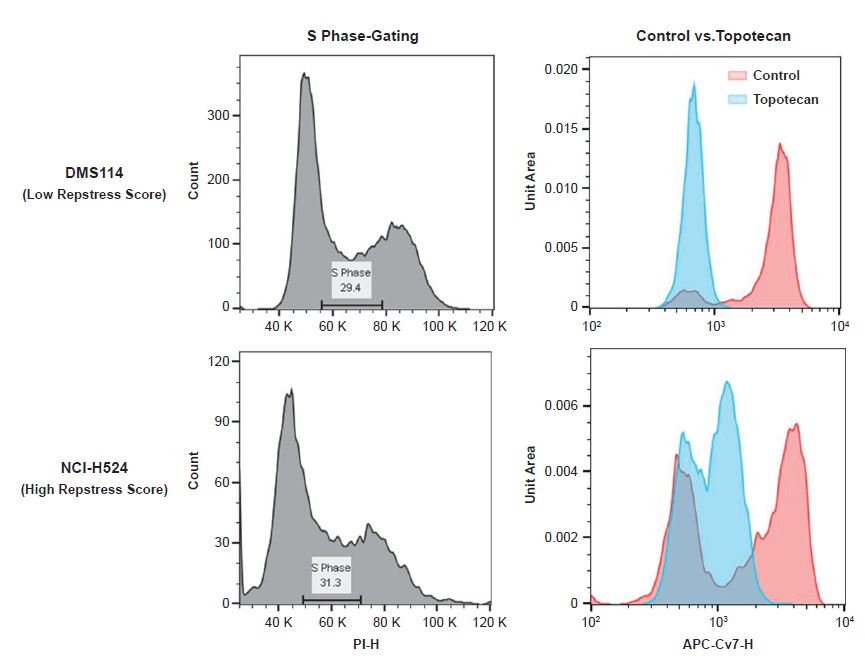

Fig. S5.**S phase gating and quantification of 5-ethynyl-2’-deoxyuridine (EdU) incorporation in small cell lung cancer (SCLC) cell lines with low (DMS11) or high (H524) repstress score
Cell cycle effects are defined by PI staining (x-axis in the left panels) and cells are labeled by EdU staining to evaluate replicating cells. Each cell line is treated with topotecan and compared EdU incorporation, shown in the right panels.
Abbreviations: PI: propidium iodide; EdU: 5-ethynyl-2’-deoxyuridine.

**
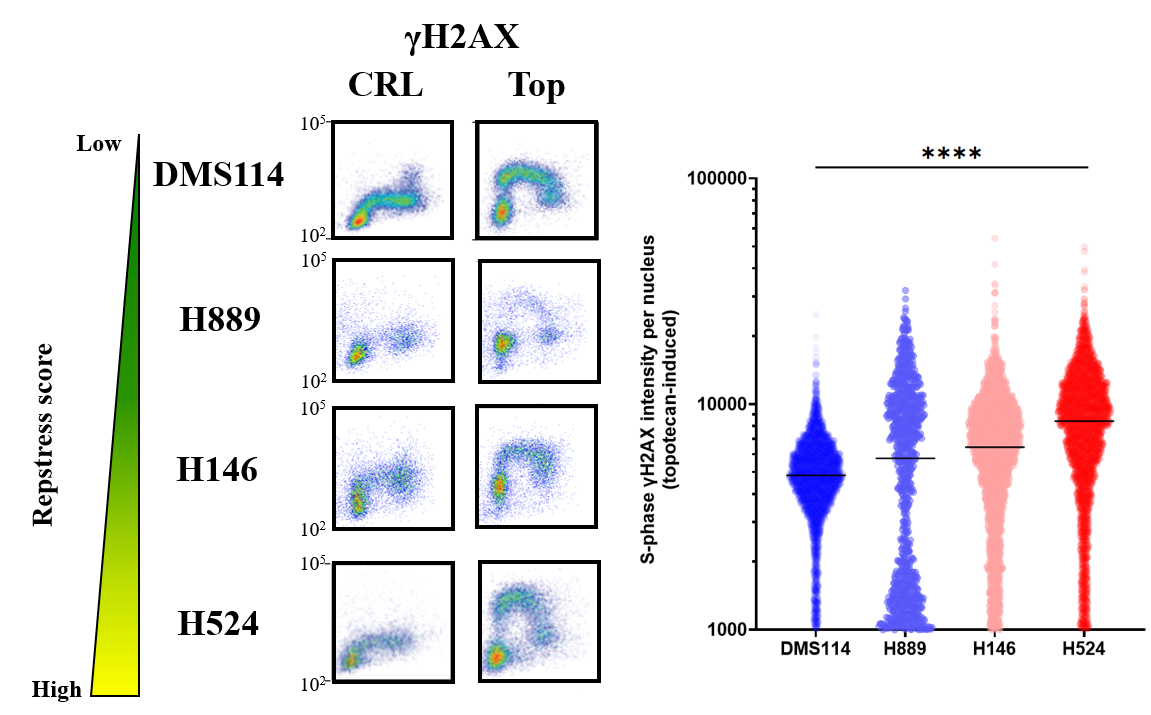
**

**Fig. S6.**Induction of γH2AX by exogenous replication stress by topotecan treatment in S phase small cell lung cancer (SCLC) cell lines
****: P < 0.0001 by linear trend test from left to right

**
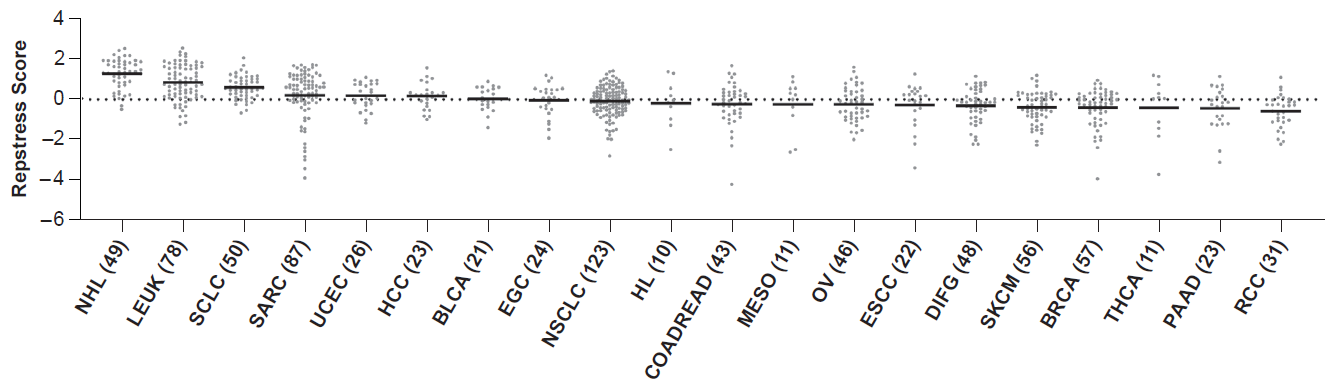

Fig. S7.**The distribution of modified repstress score excluding genes associating with neuroendocrine differentiation across 839 cancer cell lines from 20 cancer types in the CCLE
The number in the x-axis label indicates the number of tumors included in each cancer type. A dash line indicates zero of Z-normalized repstress score across all of tumors in CCLE. The black bars indicate means of Z-normalized modified repstress score within each cancer type.
Abbreviations: CCLE; Cancer Cell Line Encyclopedia; SCLC: small cell lung cancer; NHL: non-Hodgkin’s lymphoma; LEUK: leukemia; SARC: sarcoma; UCEC: uterine endometrioid cancer; EGC: esophagogastric adenocarcinoma; COADREAD: colorectal adenocarcinoma; HCC: hepatocellular carcinoma; HL: Hodgkin’s lymphoma; BLCA: bladder urothelial carcinoma; NSCLC: non-small cell lung cancer; DIFG: diffuse glioma; MESO: mesothelioma; ESCC: Esophageal squamous cell carcinoma; BRCA: breast carcinoma; THCA: Thyroid cancer; SKCM: skin melanoma; OV: ovarian cancer; PAAD: pancreatic adenocarcinoma; RCC: renal cell carcinoma.

**
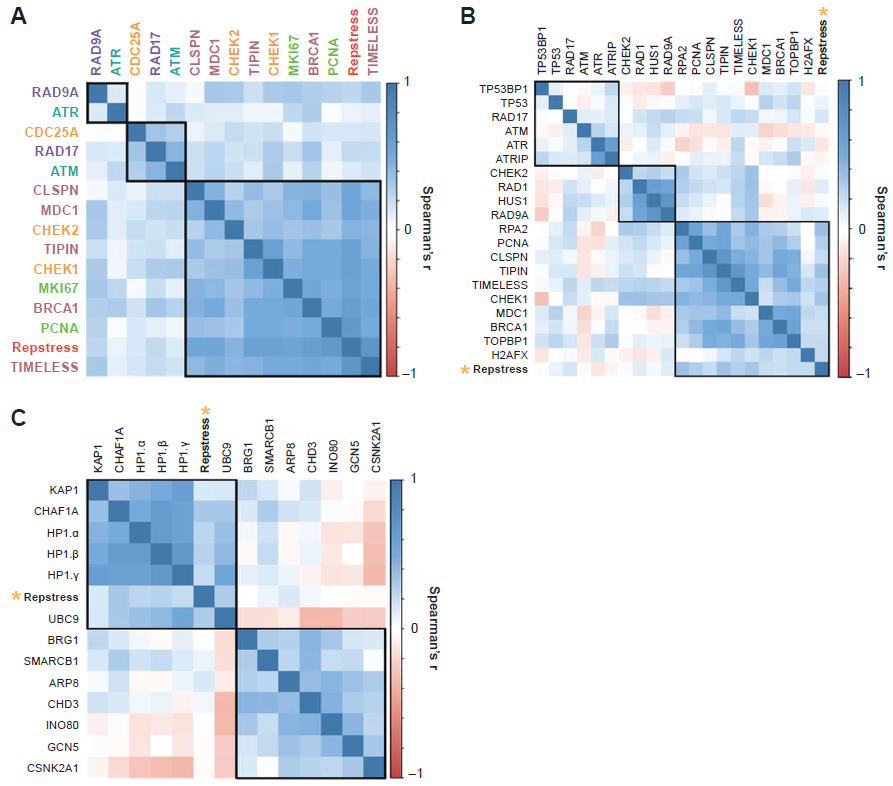

Fig. S8.**Repstress scores across distinct cancer cell lines
(**A**), Matrix of pairwise correlations between the repstress score and expressions of genes associating with replication stress-response in CCLE cancer cell lines
Colors of gene name labels denote replication stress-response functions indicated in **Fig. 1B**. Genes are clustered by Euclidean distance, using the complete-linkage clustering method, are indicated with squares with black lines.
(**B**), Pairwise correlations between repstress score and proteome expression of replication stress-response components in CCLE cancer cell lines
Proteome expression data were retrieved from a previous report (2). Genes are clustered by Euclidean distance, using the complete-linkage clustering method, are indicated with squares with black lines. Yellow stars highlight the repstress score.
(**C**), Pairwise correlations between the repstress score and gene expressions of chromatin modulators in CCLE cancer cell lines
Genes are clustered by Euclidean distance, using the complete-linkage clustering method, are indicated with squares with black lines. Yellow stars indicate the repstress score.

Abbreviations: CCLE: Cancer Cell Line Encyclopedia.

**
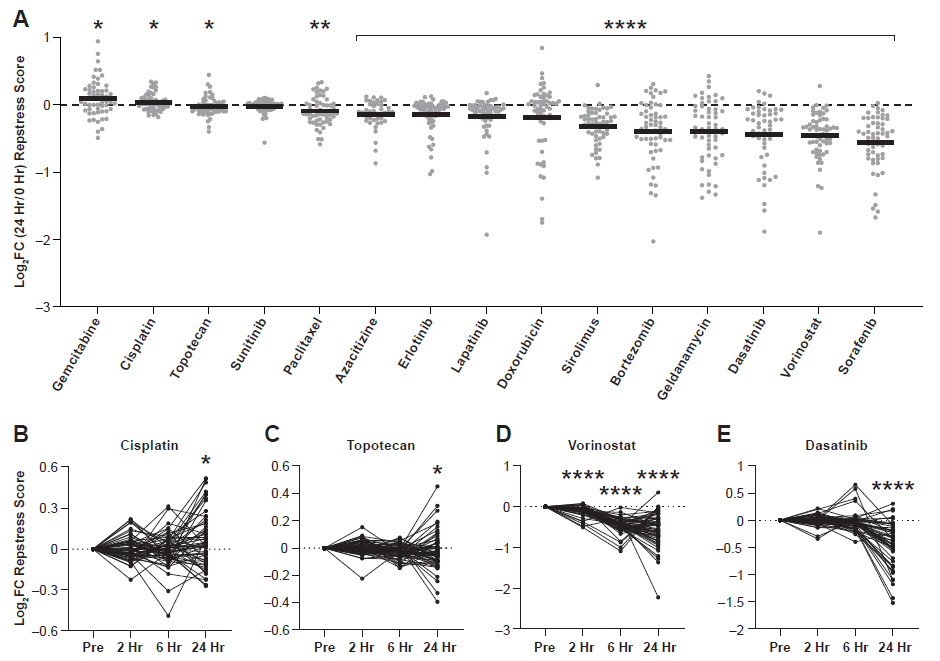

Fig. S9.**Repstress dynamics in various drugs in NCI60 cell lines
(**A**), Repstress score changes 24 hours after treatment compared with pre-treatment
Logarithm-transformed fold change of normalized repstress scores 24 hours after drug exposure in each cell line and mean are indicated with gray dots and black bars, respectively. *: P < 0.05; **: P < 0.01; ****: P < 0.0001 by Wilcoxon signed rank test comparing normalized repstress score between pre-treatment and 24 hours after treatment.
(**B**) to (**E**), Dynamics of normalized repstress score with treatment of cisplatin (B), topotecan (C), vorinostat (D), and dasatinib (E)
Repstress scores are calculated at each time point before and after treatment of indicated drugs. Repstress score in each time point is respectively divided by GI50 of each drug to normalize the contribution of decreasing proliferation due to their cytotoxicity. Data of gene expression dynamics and GI50 are publically available (9). *: P < 0.05; ****: P < 0.0001 by Wilcoxon signed rank test.
Abbreviations: Log_2_FC: log_2_-transformed fold change; GI50: concentration causing 50% cell growth inhibition; Hr: hours.

**
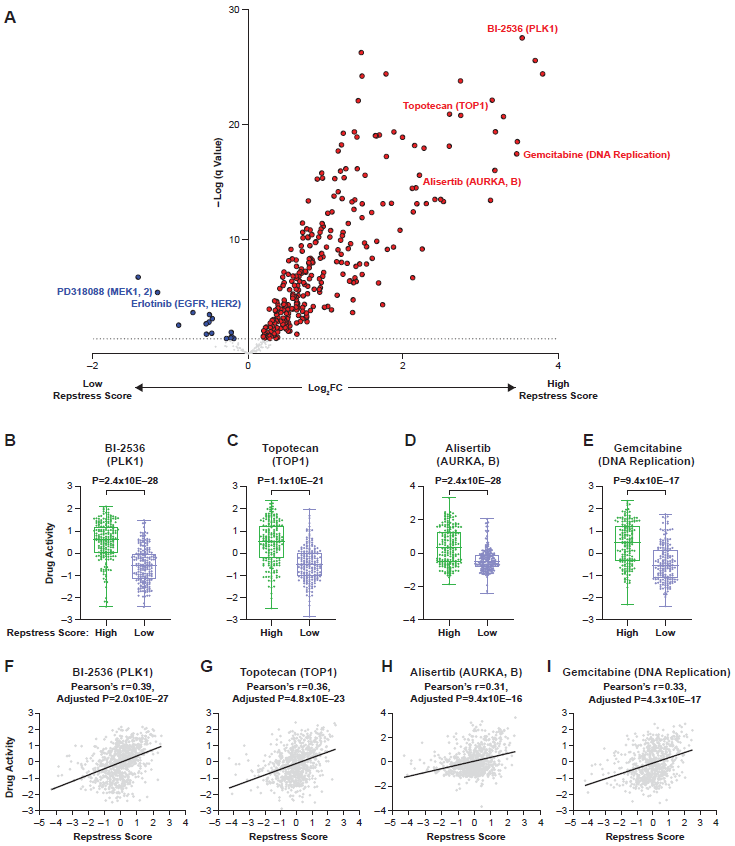

Fig. S10.**Therapeutic efficacy in CTRP cell lines with high vs. low repstress score
(**A**), A volcano plot of drug sensitivity in cancer cell lines with high vs. low repstress score
Compounds with significantly sensitive (by false discovery rate of < 5%) in cell lines with high or low repstress score are indicated with red or blue dots, respectively.
(**B**) to (**E**), Comparisons of drug activity in representative drugs (polo-like kinase-1 inhibitor BI-2536 [B], topoisomerase I inhibitor topotecan [C], aurora kinase A and B inhibitor alisertib [D], and DNA replication targeting agent gemcitabine [E]) in cancer cell lines with high vs. low repstress score
(**F**) to (**I**), Correlations between drug activity and representative drugs (polo-like kinase-1 inhibitor BI-2536 [F], topoisomerase I inhibitor topotecan [G], aurora kinase A and B inhibitor alisertib [H], and DNA replication targeting agent gemcitabine [I]) in CTRP cancer cell lines
Drug activity scores indicate calculated area under the curve over a 16-point concentration range using an automated, high-throughput workflow fitting concentration-response curves (10). High or low repstress score are defined as repstress score ≥ 75th or < 25th percentiles across CTRP cell lines, respectively. P values of each drug efficacy are initially examined by unpaired Student t test between cell lines with high vs. low repstress score across all compounds, and then adjusted for multiple testing by Benjamini-Hochberg Test for panels A to E. P values of Pearson’s correlations between repstress score and drug activity score in each drug are initially examined and then adjusted for multiple testing by Benjamini-Hochberg Test for panels F to I.
Abbreviations: CTRP: Cancer Therapeutics Response Portal; PLK1: polo-like kinase-1; TOP1: topoisomerase I; AURKA, B: aurora kinase A and B.


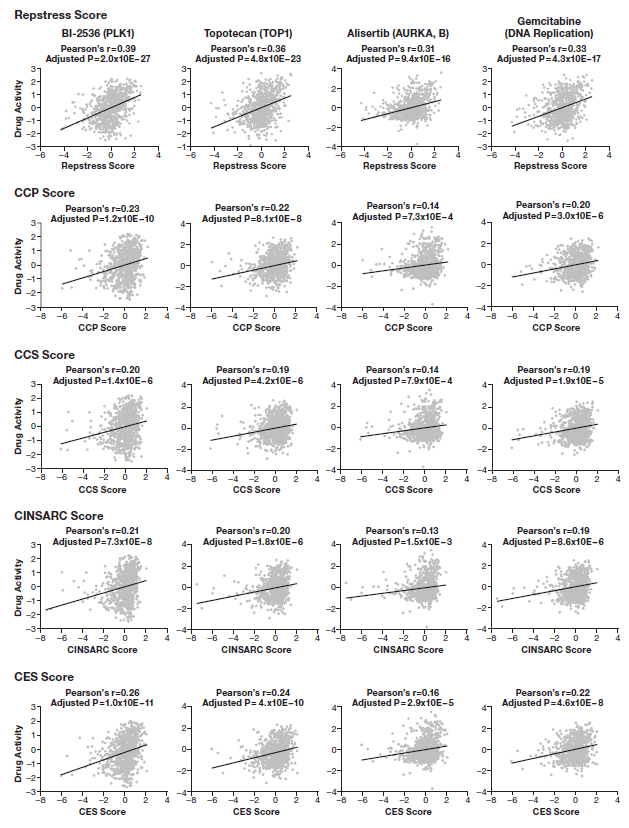


**Fig. S11.**Correlations between scores of drug activity targeting replication stress and repstress score as well as scores of previously reported cell proliferation gene signatures in CTRP cell lines
P values of Pearson’s correlations between each score and drug activity score in each drug are initially examined and then adjusted for multiple testing by Benjamini-Hochberg Test. CCP, CCS, CINSARC, and CES scores are calculated as previously reported (11-14).
Abbreviations: CTRP: Cancer Therapeutics Response Portal; CCP: cell cycle progression; CCS: cell cycle score; CINSARC: complexity index in sarcomas; CES: Centromere and kinetochore gene Expression Score


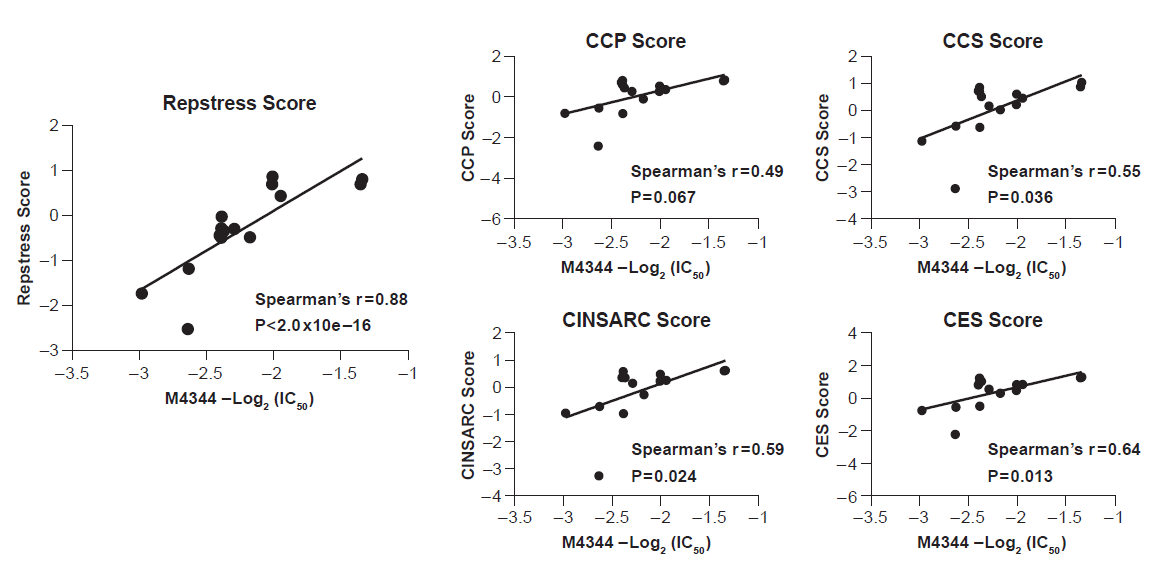


**Fig. S12.**Correlations between IC_50_ of M4344 (an ataxia telangiectasia and Rad3-related inhibitor) and repstress score as well as scores of previously reported cell proliferation gene signatures in different cancer type cell lines
The IC_50_ of M4344 in different cancer type cell lines was examined in a previous report (15). CCP, CCS, CINSARC, and CES scores were calculated as previously reported (11-14).
Abbreviations: IC_50_: half maximal inhibitory concentration; CCP: cell cycle progression; CCS: cell cycle score; CINSARC: complexity index in sarcomas; CES: Centromere and kinetochore gene Expression Score

**
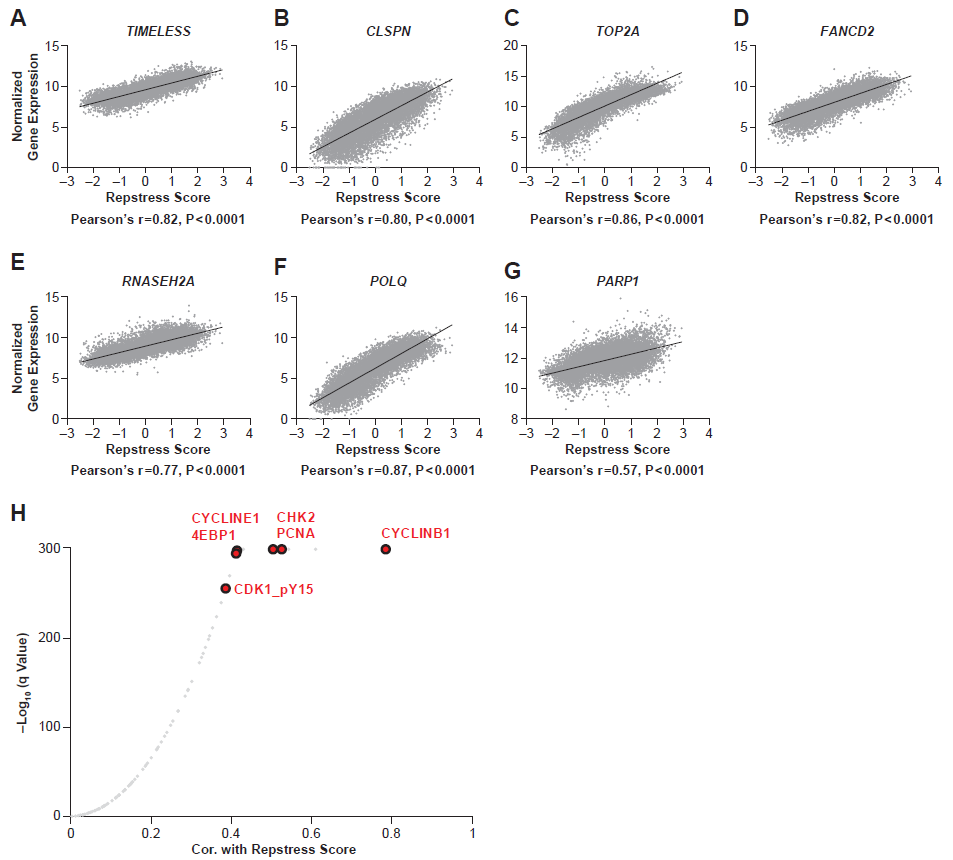

Fig. S13.**Correlations between repstress score with replication-stress response-related transcriptome and reverse phase protein array (RPPA) expression in TCGA
(**A**) to (**G**), Correlations between repstress score and replication-stress response-related gene expression (*TIMELESS* [A], *CLSPN* [B], *TOP2A* [C], *FANCD2* [D], *RNASEH2A* [E], POLQ [F], and PARP1 [G])
(**H**), A volcano plot of correlation between repstress score and reverse phase protein array (RPPA) protein expression in TCGA
Pearson’s correlations between repstress score and each RPPA expression are calculated and their p-values are adjusted using the Benjamini-Hochberg method (indicated as q value in y-axis). Representative proteins associating with replication are highlightened as red dots.
Abbreviation: Cor.: correlation coefficient.

**
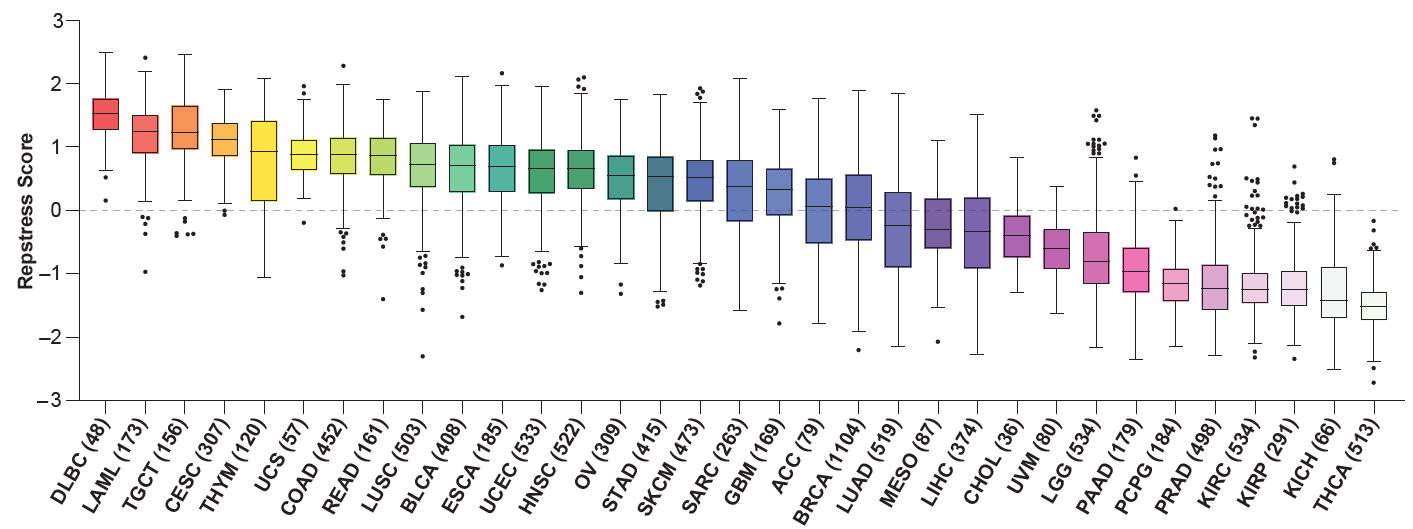

Fig. S14.**Distribution of modified repstress scores excluding genes associating with neuroendocrine differentiation across 33 cancer types in TCGA
A dash line indicates zero of Z-normalized modified repstress score across all of tumors in TCGA.
Abbreviations: TCGA: The Cancer Genome Atlas; Abbreviations for cancer types in TCGA are available in https://gdc.cancer.gov/resources-tcga-users/tcga-code-tables/tcga-study-abbreviations.

**
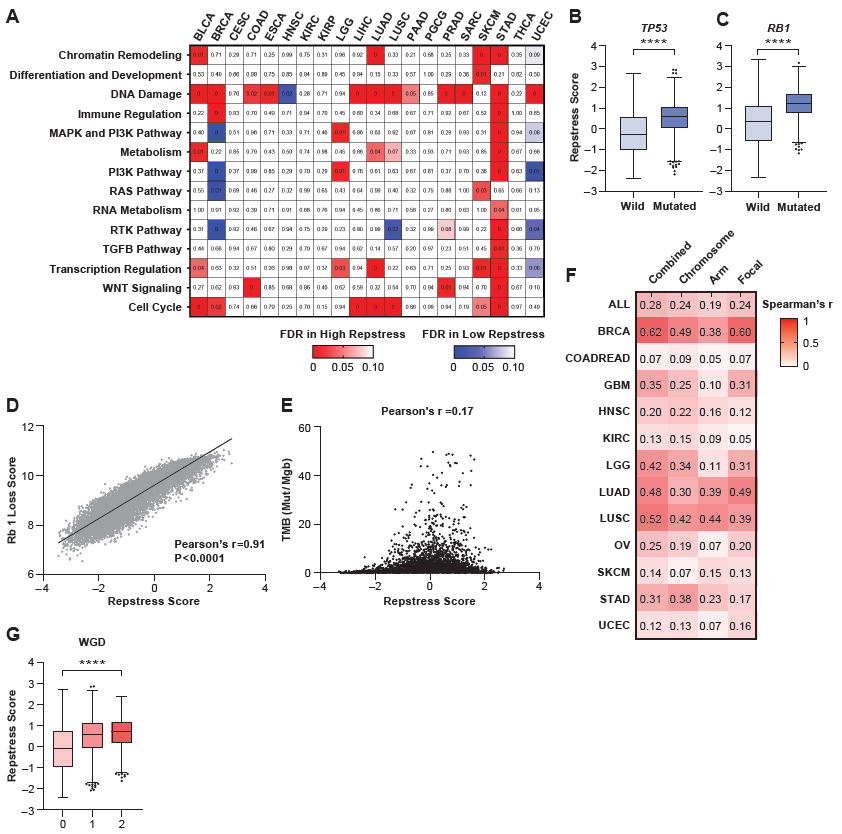

Fig. S15.**Associations between genomic alteration and repstress score in TCGA
(**A**), Heatmap of false discovery rate comparing repstress scores between TCGA tumors with and without indicated pathway gene mutations in each cancer type
False discovery rate is calculated by Mann-Whitney U test comparing repstress score between tumors with mutations in indicated pathways vs. those without, followed by Benjamini-Hochberg method. The number in each column indicates adjusted P value, with 0 indicates adjusted P value of < 0.0001. Red or blue denote instances in which pathway-mutated samples have higher or lower repstress scores, respectively. Pathway-associated genes are retrieved from a previous report (16). Cancer types with n < 150 are excluded. Hypermutated tumors (silent point mutational burden ≥ 50 mutation per megabase) are excluded from this analysis.
(**B**), (**C**), Comparisons of repstress scores between *TP53* (B) or *RB1* (C) mutated and non-mutated tumors
****: P < 0.0001 by unpaired Student’s t test.
(**D**), A correlation between repstress score and Rb1 loss signature score in TCGA
Rb1 loss signature is described in a previous report (17).
(**E**), A scatter plot between repstress score and TMB of silent point mutations for TCGA tumor samples
Hypermutated tumors (i.e. mutational burden of ≥ 50 mutations per megabase) are excluded.
(**F**), Correlation matrix of chromosome, arm, focal, and combined somatic copy number alteration (SCNA) scores with repstress score in different cancer types
Combined somatic copy number scores in TCGA are available in a previous report (16). Number in each column indicates Spearman’s correlation coefficient.
(**G**), A comparison of repstress score among tumors divided by whole genome doubling (WGD) levels
Levels of whole genome doubling are retrieved from a previous report (18). ****: P < 0.0001 by linear trend test from left to right.
Abbreviations: TCGA: The Cancer Genome Atlas; FDR: false discovery rate; WGD: whole genome doubling. Abbreviations for cancer types in TCGA are available in https://gdc.cancer.gov/resources-tcga-users/tcga-code-tables/tcga-study-abbreviations.

**
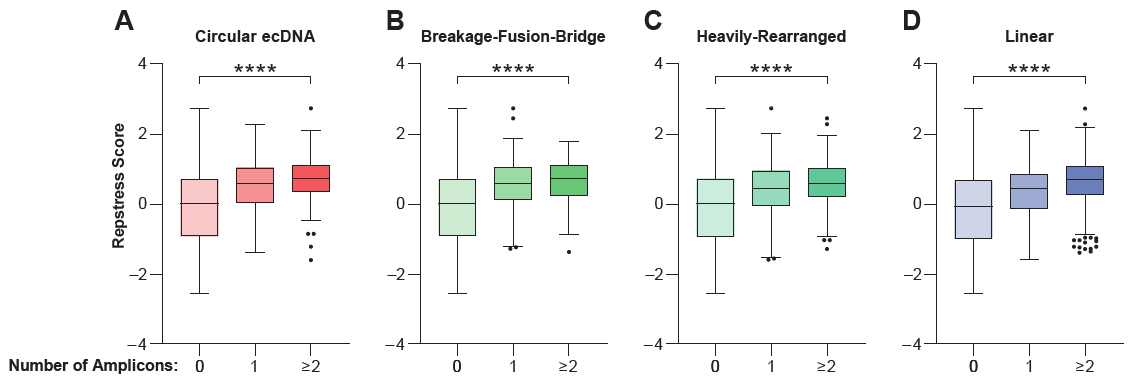

Fig. S16.**Comparisons of repstress score in TCGA tumors with extra-chromosomal DNA (ecDNA) amplification
The numbers of each amplicon in TCGA tumors are retrieved from a previous report (6). ****: P < 0.0001 by linear trend test from left to right.
Abbreviations: TCGA: The Cancer Genome Atlas; ecDNA: extrachromosomal DNA

**
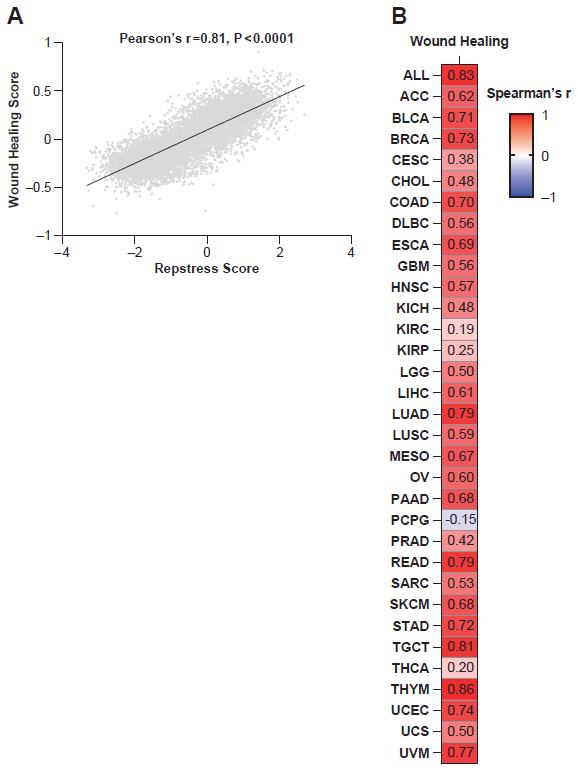

Fig. S17.**Correlations between repstress score and wound healing score in TCGA
(**A**), Correlation between repstress score and wound healing score in TCGA
Wound healing score is derived by gene signature-based approach in TCGA (19).
(**B**), Spearman’s correlations between repstress score and wound healing score across cancer types in TCGA
The numbers in each column indicate Spearman’s coefficient (r).
Abbreviations: TCGA: The Cancer Genome Atlas; abbreviations for cancer types in TCGA are available in https://gdc.cancer.gov/resources-tcga-users/tcga-code-tables/tcga-study-abbreviations.

**
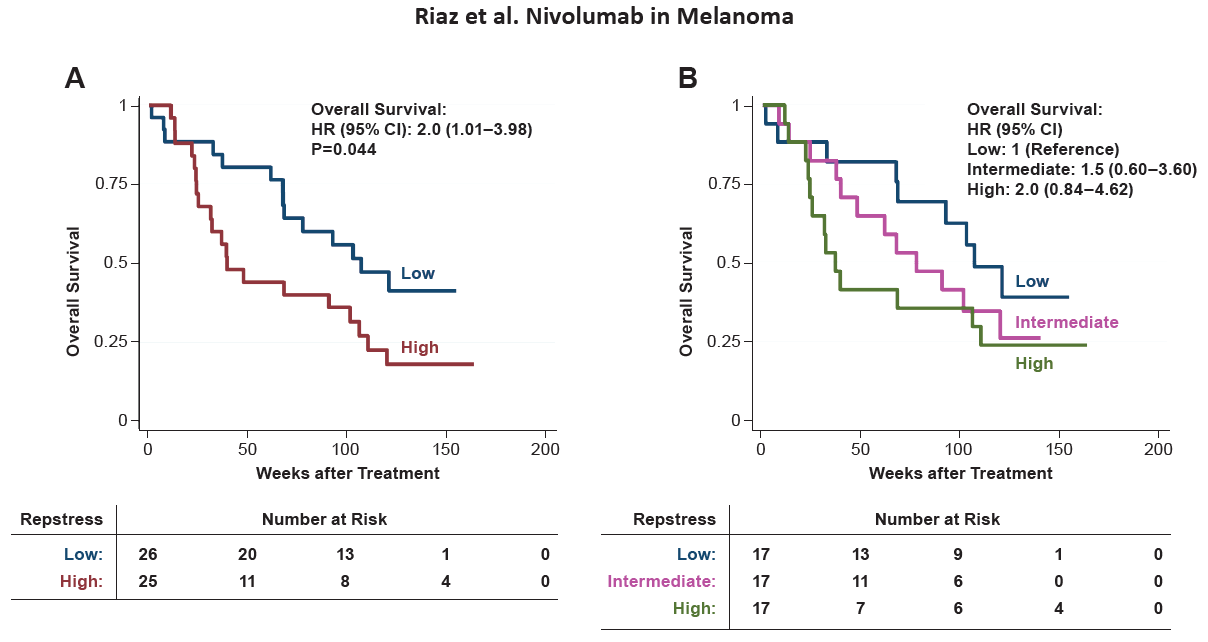

Fig. S18.**Differences of overall survival by repstress score in melanoma patients treated with nivolumab
(**A**), Overall survival in melanoma patients with high vs. low repstress score
High vs. low repstress scores are defined as patients whose cancers have repstress score ≥ or < median in an independent dataset (7), respectively. P value is derived from the log-rank test.
(**B**), Overall survival in melanoma patients with high vs. intermediate vs. low repstress score
High, intermediate, low repstress score are defined as patients whose cancers have repstress score in highest tertile, 2nd tertile, and 3rd tertile in an independent dataset (7), respectively.
Abbreviations: HR: hazard ratio; CI: confidence interval.

**
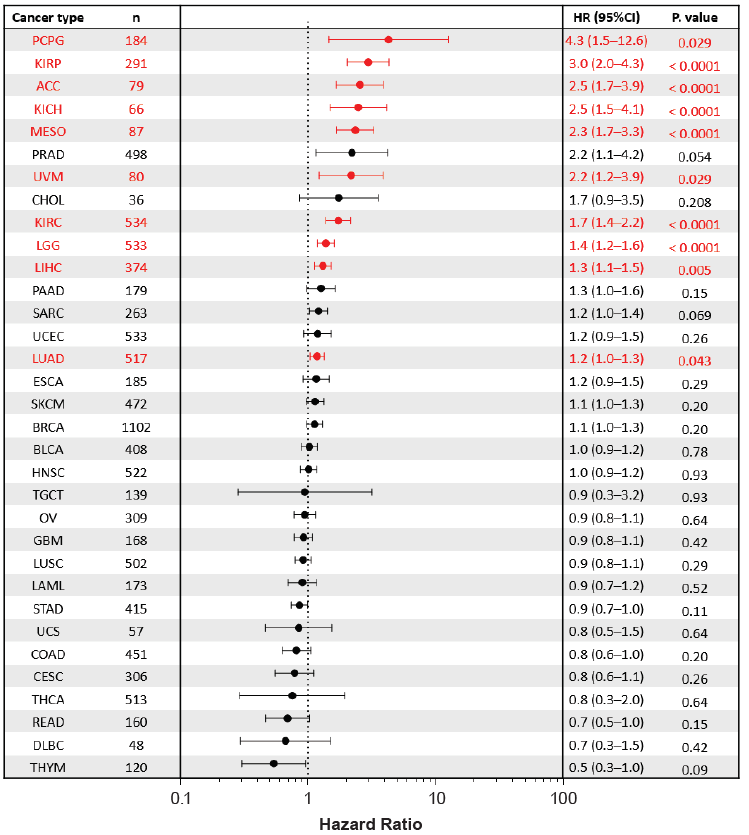

Fig. S19.**Contribution of repstress score to overall survival within each cancer type in TCGA
The forest plot indicates the results of Cox proportion hazard analysis using repstress score as a continuous value within each cancer type. The forest plot shows logarithm-scaled hazard ration and their 95% confidence intervals with circles and lines, respectively. P values of Cox proportion hazards analysis are adjusted by Benjamini-Hochberg method and indicated on the right. Cancer types with adjusted P value of < 0.05 are highlightened with red.
Abbreviations: TCGA: The Cancer Genome Atlas; HR: hazard ratio; CI: confidence interval. Abbreviations for cancer types in TCGA are available in https://gdc.cancer.gov/resources-tcga-users/tcga-code-tables/tcga-study-abbreviations.

**
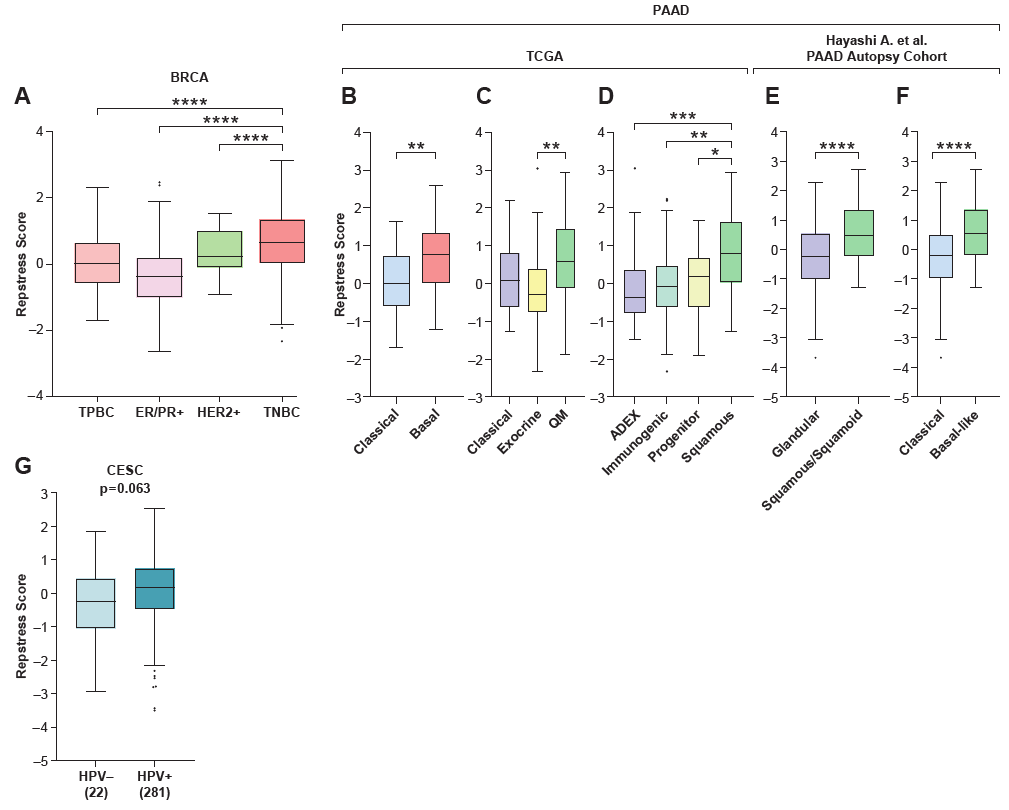

Fig. S20.**Repstress score comparisons in distinct histological and molecular subtypes of particular cancer types
(**A**), Comparison of repstress score across different targetable receptor-expressing subtypes in breast cancer
****: P < 0.0001 by one-way ANOVA followed by Tukey’s multiple comparison test.
(**B**) to (**D**), Comparisons of repstress score across transcriptomic molecular subtypes by Moffitt et al. (20) (B), Collisson et al. (21) (C), and Bailey et al. (22) (D) in TCGA pancreatic cancer.
*: P < 0.05, **: P < 0.01, ***: P < 0.001 by Mann-Whitney U test in (B) and One-way ANOVA followed by Tukey’s multiple comparison test in (C) and (D). Annotations of histological and molecular subtypes are retrieved from a previous report (8).
(**E**), (**F**), Comparisons of repstress score across histological (E) and transcriptomic (F) phenotypes in autopsy samples from patients with pancreatic cancer
****: P < 0.0001 by Mann-Whitney U test. Gene expressions and histological and molecular subtypes are retrieved from a previous report (8).
(**G**), Comparison of repstress scores between patients with HPV-null (HPV-) or HPV-driven (HPV+) cervical cancers
Numbers with HPV status at bottom indicate the number of tumors included. P value is derived from Mann-Whitney U test.
Abbreviations: TCGA: The Cancer Genome Atlas; TPBC: triple positive breast cancer; ER: estrogen receptor; PR: progesterone receptor; HER2: human epidermal growth factor receptor 2; TNBC: triple negative breast cancer; QM: Quasi-mesenchymal; ADEX: aberrantly differentiated endocrine exocrine; HPV: human papilloma virus; Abbreviations for cancer types in TCGA are available in https://gdc.cancer.gov/resources-tcga-users/tcga-code-tables/tcga-study-abbreviations.

**
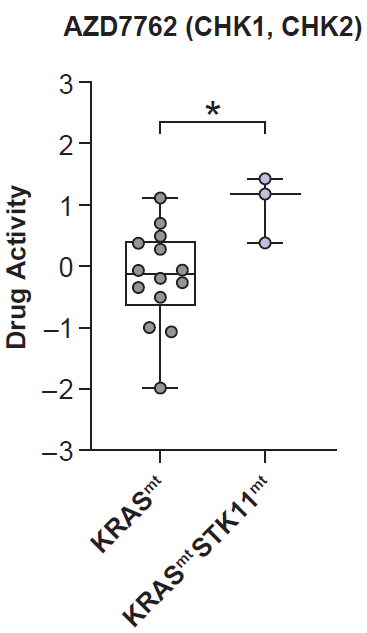

Fig. S21.**Drug activity of a CHK1/2 inhibitor AZD7762 in *KRAS*/*STK11* co-mutated non-small cell lung cancer cell lines in GDSC
Cell lines with co-mutation in *KRAS*/*TP53* or *KRAS*/*CDKN2A* are excluded from this analysis given these cell lines are considered as distinct biology from *KRAS*/*STK11* co-mutated or *KRAS* single-mutated non-small cell lung cancer. Drug activity score indicates negative logarithm-transformed half maximal inhibitory concentration (molar) and is retrieved from CellMiner CDB (23) https://discover.nci.nih.gov/cellminercdb/. Dots, box, and ranges indicate drug activity scores in each cell lines, median/quartile range, and maximum/minimum in non-small cell lung cancer cell lines with indicated gene alterations, respectively. *: P < 0.05 by Mann-Whitney U test.
Abbreviations: STK11: Serine/threonine kinase 11; GDSC: Genomics of Drug Sensitivity in Cancer; mt: mutated.

**Table S1.**Repstress genes, their weights to calculate the score, and their function

| **Genes** | **Weight** | **Function** |
| --- | --- | --- |
| AURKB | 0.24010608 | These kinases participate in the regulation of alignment and segregation of chromosomes during mitosis and meiosis through association with microtubules. |
| CCNA2 | 0.68259341 | The protein encoded by this gene belongs to the highly conserved cyclin family, whose members function as regulators of the cell cycle. |
| GADD45G | 0.36582618 | A member of a group of genes whose transcript levels are increased following stressful growth arrest conditions and treatment with DNA-damaging agents. The protein encoded by this gene responds to environmental stresses by mediating activation of the p38/JNK pathway via MTK1/MEKK4 kinase. |
| GINS1 | 0.6298731 | The formation of the GINS complex is essential for the initiation of DNA replication in yeast and Xenopus egg extracts |
| LIG3 | 0.49944914 | A member of the DNA ligase family. Each member of this family encodes a protein that catalyzes the joining of DNA ends |
| MTF2 | 0.36441812 | Metal response element binding transcription factor 2 |
| ORC6 | 0.70672691 | The origin recognition complex (ORC) is a highly conserved six subunit protein complex essential for the initiation of the DNA replication in eukaryotic cells. |
| POLA1 | 0.68584521 | This gene encodes the catalytic subunit of DNA polymerase, which together with a regulatory and two primase subunits, forms the DNA polymerase alpha complex. The catalytic subunit plays an essential role in the initiation of DNA replication. |
| POLD4 | -0.71984532 | This gene encodes the smallest subunit of DNA polymerase delta. DNA polymerase delta possesses both polymerase and 3' to 5' exonuclease activity and plays a critical role in DNA replication and repair. The encoded protein enhances the activity of DNA polymerase delta and plays a role in fork repair and stabilization through interactions with the DNA helicase Bloom syndrome protein. Alternatively spliced transcript variants encoding multiple isoforms have been observed for this gene |
| POLE4 | -0.07612991 | POLE4 is a histone-fold protein that interacts with other histone-fold proteins to bind DNA in a sequence-independent manner. These histone-fold protein dimers combine within larger enzymatic complexes for DNA transcription, replication, and packaging. |
| PRPS1 | 0.56064911 | An enzyme that catalyzes the phosphoribosylation of ribose 5-phosphate to 5-phosphoribosyl-1-pyrophosphate, which is necessary for purine metabolism and nucleotide biosynthesis |
| RFC5 | 0.47485085 | This gene encodes the smallest subunit of the replication factor C complex, which consists of five distinct subunits (140, 40, 38, 37, and 36 kDa) and is required for DNA replication. This subunit interacts with the C-terminal region of proliferating cell nuclear antigen and is required to open and load proliferating cell nuclear antigen onto DNA during S phase. It is a member of the AAA+ (ATPases associated with various cellular activities) ATPase family and forms a core complex with the 38 and 40 kDa subunits that possesses DNA-dependent ATPase activity. |
| RMI1 | 0.55448942 | RMI1 is a component of protein complexes that limit DNA crossover formation via the dissolution of double Holliday junctions |
| RRM1 | 0.68108176 | This gene encodes the large and catalytic subunit of ribonucleotide reductase, an enzyme essential for the conversion of ribonucleotides into deoxyribonucleotides. A pool of available deoxyribonucleotides is important for DNA replication during S phase of the cell cycle as well as multiple DNA repair processes. Alternative splicing results in multiple transcript variants |
| SRSF1 | 0.68007925 | a member of the arginine/serine-rich splicing factor protein family. The encoded protein can either activate or repress splicing, depending on its phosphorylation state and its interaction partners |
| SUV39H1 | 0.59562491 | The encoded protein is a histone methyltransferase that trimethylates lysine 9 of histone H3, which results in transcriptional gene silencing |
| TNPO2 | 0.5009049 | Transportin 2 protein (nuclear – cytoplasm transporter) |

**Table S2.**Multivariate Cox regression analysis of overall survival in TCGA

| **Factors** | **Hazard**  **ratio** | **Standard**  **error** | **P value** | **95%**  **Confidence Interval** | |
| --- | --- | --- | --- | --- | --- |
| Age at diagnosis (years) | 1.02 | 0.01 | <0.0001 | 1.02 | 1.03 |
| Female  (ref. male = 1) | 1.00 | 0.05 | 0.96 | 0.91 | 1.09 |
| Pathological/clinical stage (ref. Stage I = 1) |  |  |  |  |  |
| Stage II | 1.51 | 0.09 | <0.0001 | 1.34 | 1.71 |
| Stage III | 2.45 | 0.15 | <0.0001 | 2.18 | 2.75 |
| Stage IV | 4.63 | 0.32 | <0.0001 | 4.04 | 5.31 |
| Repstress score | 1.01 | 0.005 | 0.039 | 1 | 1.02 |

This analysis is also adjusted by cancer types (n=33) as categorical values.
Abbreviations: TCGA: The Cancer Genome Atlas; ref: reference.

**References**

1. Ghandi M, Huang FW, Jané-Valbuena J, Kryukov GV, Lo CC, McDonald ER, 3rd*, et al.* Next-generation characterization of the Cancer Cell Line Encyclopedia. Nature **2019**;569:503-8

2. Nusinow DP, Szpyt J, Ghandi M, Rose CM, McDonald ER, 3rd, Kalocsay M*, et al.* Quantitative Proteomics of the Cancer Cell Line Encyclopedia. Cell **2020**;180:387-402.e16

3. Beane JE, Mazzilli SA, Campbell JD, Duclos G, Krysan K, Moy C*, et al.* Molecular subtyping reveals immune alterations associated with progression of bronchial premalignant lesions. Nature communications **2019**;10:1856

4. Hoadley KA, Yau C, Hinoue T, Wolf DM, Lazar AJ, Drill E*, et al.* Cell-of-Origin Patterns Dominate the Molecular Classification of 10,000 Tumors from 33 Types of Cancer. Cell **2018**;173:291-304.e6

5. Marx V. Drilling into big cancer-genome data. Nature methods **2013**;10:293-7

6. Kim H, Nguyen NP, Turner K, Wu S, Gujar AD, Luebeck J*, et al.* Extrachromosomal DNA is associated with oncogene amplification and poor outcome across multiple cancers. Nature genetics **2020**;52:891-7

7. Riaz N, Havel JJ, Makarov V, Desrichard A, Urba WJ, Sims JS*, et al.* Tumor and Microenvironment Evolution during Immunotherapy with Nivolumab. Cell **2017**;171:934-49.e16

8. Hayashi A, Fan J, Chen R, Ho Y-j, Makohon-Moore AP, Lecomte N*, et al.* A unifying paradigm for transcriptional heterogeneity and squamous features in pancreatic ductal adenocarcinoma. Nature Cancer **2020**;1:59-74

9. Monks A, Zhao Y, Hose C, Hamed H, Krushkal J, Fang J*, et al.* The NCI Transcriptional Pharmacodynamics Workbench: A Tool to Examine Dynamic Expression Profiling of Therapeutic Response in the NCI-60 Cell Line Panel. Cancer research **2018**;78:6807-17

10. Rees MG, Seashore-Ludlow B, Cheah JH, Adams DJ, Price EV, Gill S*, et al.* Correlating chemical sensitivity and basal gene expression reveals mechanism of action. Nature chemical biology **2016**;12:109-16

11. Cuzick J, Swanson GP, Fisher G, Brothman AR, Berney DM, Reid JE*, et al.* Prognostic value of an RNA expression signature derived from cell cycle proliferation genes in patients with prostate cancer: a retrospective study. The Lancet Oncology **2011**;12:245-55

12. Lundberg A, Lindström LS, Harrell JC, Falato C, Carlson JW, Wright PK*, et al.* Gene Expression Signatures and Immunohistochemical Subtypes Add Prognostic Value to Each Other in Breast Cancer Cohorts. Clinical cancer research : an official journal of the American Association for Cancer Research **2017**;23:7512-20

13. Chibon F, Lagarde P, Salas S, Pérot G, Brouste V, Tirode F*, et al.* Validated prediction of clinical outcome in sarcomas and multiple types of cancer on the basis of a gene expression signature related to genome complexity. Nature medicine **2010**;16:781-7

14. Zhang W, Mao JH, Zhu W, Jain AK, Liu K, Brown JB*, et al.* Centromere and kinetochore gene misexpression predicts cancer patient survival and response to radiotherapy and chemotherapy. Nature communications **2016**;7:12619

15. Jo U, Senatorov IS, Zimmermann A, Saha LK, Murai Y, Kim SH*, et al.* Novel and Highly Potent ATR Inhibitor M4344 Kills Cancer Cells With Replication Stress, and Enhances the Chemotherapeutic Activity of Widely Used DNA Damaging Agents. Mol Cancer Ther **2021**;20:1431-41

16. Davoli T, Uno H, Wooten EC, Elledge SJ. Tumor aneuploidy correlates with markers of immune evasion and with reduced response to immunotherapy. Science (New York, NY) **2017**;355

17. Ertel A, Dean JL, Rui H, Liu C, Witkiewicz AK, Knudsen KE*, et al.* RB-pathway disruption in breast cancer: differential association with disease subtypes, disease-specific prognosis and therapeutic response. Cell cycle (Georgetown, Tex) **2010**;9:4153-63

18. Taylor AM, Shih J, Ha G, Gao GF, Zhang X, Berger AC*, et al.* Genomic and Functional Approaches to Understanding Cancer Aneuploidy. Cancer cell **2018**;33:676-89.e3

19. Thorsson V, Gibbs DL, Brown SD, Wolf D, Bortone DS, Ou Yang TH*, et al.* The Immune Landscape of Cancer. Immunity **2018**;48:812-30.e14

20. Moffitt RA, Marayati R, Flate EL, Volmar KE, Loeza SG, Hoadley KA*, et al.* Virtual microdissection identifies distinct tumor- and stroma-specific subtypes of pancreatic ductal adenocarcinoma. Nature genetics **2015**;47:1168-78

21. Collisson EA, Sadanandam A, Olson P, Gibb WJ, Truitt M, Gu S*, et al.* Subtypes of pancreatic ductal adenocarcinoma and their differing responses to therapy. Nature medicine **2011**;17:500-3

22. Bailey P, Chang DK, Nones K, Johns AL, Patch AM, Gingras MC*, et al.* Genomic analyses identify molecular subtypes of pancreatic cancer. Nature **2016**;531:47-52

23. Rajapakse VN, Luna A, Yamade M, Loman L, Varma S, Sunshine M*, et al.* CellMinerCDB for Integrative Cross-Database Genomics and Pharmacogenomics Analyses of Cancer Cell Lines. iScience **2018**;10:247-64
